# The Effect of Network-Level Payment Models on Care Network Performance: A Scoping Review of the Empirical Literature

**DOI:** 10.5334/ijic.6002

**Published:** 2022-04-01

**Authors:** Thomas Reindersma, Sandra Sülz, Kees Ahaus, Isabelle Fabbricotti

**Affiliations:** 1 Health Services Management & Organisation, Erasmus School of Health Policy & Management, Erasmus University Rotterdam, Rotterdam, The Netherlands

**Keywords:** health care networks, alternative payment models, performance, global payments, network reimbursement

## Abstract

**Introduction:**

Traditional payment models reward volume rather than value. Moving away from reimbursing separate providers to network-level reimbursement is assumed to support structural changes in health care organizations that are necessary to improve patient care. This scoping review evaluates the performance of care networks that have adopted network-level payment models.

**Methods:**

A scoping review of the empirical literature was conducted according to the five-step York framework. We identified indicators of performance, categorized them in four categories (quality, utilization, spending and other consequences) and scored whether performance increased, decreased, or remained stable due to the payment model.

**Results:**

The 76 included studies investigated network-level capitation, disease-based bundled payments, pay-for-performance and blended global payments. The majority of studies stem from the USA. Studies generally concluded that performance in terms of quality and utilization increased or remained stable. Most payment models were associated with improved spending performance. Overall, our review shows that network-level payment models are moderately successful in improving network performance.

**Discussion/conclusion:**

As health care networks are increasingly common, it seems fruitful to continue experimenting with reimbursement models for health care networks. It is also important to broaden the scope to not only scrutinize outcomes, but also the contexts and mechanisms that lead to certain outcomes.

## Introduction

Fragmented health care leads to poor system and patient outcomes. Fragmentation manifests itself in a myriad of ways, such as duplication of services and lack of involvement, ownership, or communication [1]. Ageing populations and multi-morbidity amplify these issues, making it more relevant to address fragmentation. In order to do so, governments and policymakers increasingly rely on networks of health care organizations [2,3]. As an alternative to market or quasi-market structures, networks enable separate health care entities to work together and coordinate care [4,5]. However, the current ways of paying for care seem to impede coordination within networks. Providers are predominantly reimbursed separately, through traditional payment models such as fee-for-service (FFS) or diagnosis-related-groups (DRGs), leaving the paywalls between organizations intact [6]. It is widely assumed that most traditional models reward volume [7], discourage prevention [8], impede care coordination [7], and stimulate delivering the most profitable services [9]. In essence, traditional models are perceived as not being able to create the right incentives for the integration of care, leading instead to an array of misaligned incentives [10]. Moving away from separate provider reimbursement to network-level reimbursement would support interorganizational coordination, flexible use of resources between organizations, and innovation in delivery design and IT [11,12,13]. Subsequently, it is assumed that developing adequate network-level payment models is essential to achieving high-quality patient care. Health care purchasers, policymakers and providers have correspondingly initiated demonstrations and experiments with novel network-level payment models. However, to date, how these payment models contribute to network performance has not been systematically investigated.

The current study adds to previous research by considering all payment models that are aimed specifically at joint reimbursement of networks. Although previous reviews have focused on various subsets of payment models, these reviews have not made a primary distinction between disbursement to a network and to separate providers. For example, Cattel and Eijkenaar [8] focused on key design features of value-based payment (VBP) initiatives and included 24 papers that shed light on VPB effects, but on the initiative level rather than payment model level. Vlaanderen et al. [14] conducted an analysis of the characteristics of outcome-based payment (OBP) models and their effects in terms of structure, process, and outcome indicators. Kaufman et al. [15] provide an overview of utilization, care, and outcomes associated with accountable care organizations (ACOs) in the USA. Thus, VBP, OBP, and ACO models have been systematically reviewed separately, but an overview of all network-level payment models, transcending definitions of VBP, OBP, and ACO models, and their performance, is lacking. Our aim is to study how such network-level payment models affect the performance of networks. We summarize this in the following research question: what is the effect of network-level payment models on the performance of care networks? From the resulting comprehensive overview of performance indicators, policymakers and health care professionals can, depending on what performance indicators they deem important, make a more informed decision when implementing a network-level payment model.

## Theoretical Framework

### Payment Models, Networks, and Performance

Payment models refer to the funding mechanisms that health care purchasers adopt in order to financially reimburse providers of care or, in this case, care networks. The term network-level payment model is used to indicate a payment model in which a set of providers or facilities are jointly reimbursed through a contracting entity (i.e., the network or one network provider), which in turn can then disburse the money received to the providers in the care network. Care networks are defined as sets of two or more legally autonomous providers [see 16] that are tasked with the coordination of care pathways and the execution of clinical interventions across providers [5]. The term provider is used to denote a practice, hospital, or other setting, and not an individual physician, unless otherwise noted. Network performance is defined as the ability of the network to satisfy the payment model’s objectives as made explicit in the included studies. In our study, the taxonomy of payment models by Tsiachristas [17] has been used to identify and categorize network-level payment models (henceforth referred to as payment models). Non-network-level models have been excluded from this taxonomy (see ***Table 1***) as they are not the focus of our study.

**Table 1:** Taxonomy of network-level payment models, adapted from Tsiachristas [17].


PAYMENT MODEL	DEFINITION

***Base payment***	

Capitation	Periodic lump sum per enrolled patient for a range of services

Episode-based bundled payment	Payment for medical services delivered during an episode of care

Disease-based bundled payment	Payment for all the care required by a patient for a particular disease over a predefined period

Global payment	Payment for all the services offered to cover the medical needs of a defined population for a specific period of time

***Add-on payment***	

Pay-for-performance (P4P)	Payments to providers for meeting predetermined performance indicators

Pay-for-coordination (P4C)	Payment for taking responsibility for coordinating a patient’s care along parts of, or complete, care pathways for a specific period

Risk and gain sharing/Shared savings	Payments are increased if financial targets are met for the wider system/Providers share in savings and losses if financial or quality targets are (not) met


### Intended and Unintended Consequences of Payment Models

How payment models incentivize structural change will depend on the payment model. It is assumed that, given the appropriate incentives, providers will be able to deliver the right care at the right time in the right way, and at the right place [18,19]. Under a capitation system, providers receive a periodic lump sum per enrolled patient for a defined set of services. This incentivizes providers to minimize costs, thereby encouraging them to innovate in cost-reducing technologies, select lower-cost alternative treatments, and invest in prevention. The downsides are increased financial risk for providers, and the temptation to stint on care and avoid high-risk patients, often referred to as ‘cherry picking’ [13,20]. Episode-based bundled payments cover medical services delivered during an episode of care. Providers are thereby encouraged to coordinate and organize care activities within an episodic bundle to eliminate unnecessary and expensive care and reduce costs [7]. However, there is little incentive to avoid unnecessary episodes [12] since more care episodes implies more revenue. Disease-based bundled payments have a broader scope, covering all the care required for a patient with a particular disease during a predefined period. As with episode-based bundled payments, coordination between providers is encouraged. Providers are incentivized to improve quality since they bear the financial burden of complications and avoidable services, such as hospital readmissions. For both bundled payment types, costs incurred that exceed the pre-agreed payment are at the expense of the provider and similarly if the costs are less than the payment, providers retain the difference. This approach may lead to stinting on care and cherry picking if adequate quality monitoring is not in place, and patient choice might be limited due to a limited and fixed provider set [12]. In another approach, a global payment is made to cover all medical services for a defined population during a period of time. In the literature, this term is used interchangeably with population-based payment and global budgets. A global payment model shares some properties with bundled payments and capitation but can offer greater managerial flexibility in allocating resources and enables innovation in delivery design [12,13]. A specific downside of global payments is that population health might be prioritized above individual health [12].

These basic payment models are often enhanced with additional payment formula: pay-for-performance (P4P), pay-for-coordination (P4C), risk and gain sharing and shared savings. Risk sharing arrangements, such as risk-and-gain-sharing and shared savings, are intended to increase efficiency in care delivery [20]. In part, this works through weakening the providers’ tendencies to overtreat patients [21]. Payers or providers can decide whether to agree to one-sided risk only (upside risk) or two-sided risk (upside and downside risk) and can also tweak the percentages of savings and losses that are shared [22]. In a one-sided risk arrangement, providers share only in gains, whereas in a two-sided risk arrangement gains and losses are both shared. Loss aversion theory argues that losses have a stronger psychological effect than have gains [23]. This implies that a two-sided risk arrangement will more strongly incentivize providers, and so have the potential to enhance performance. Providers that want to benefit from shared savings will have to improve in terms of quality and cost measures [24]. All the above payment models are risk-based, except for P4P and P4C. If employing P4P, providers receive a payment for meeting predetermined performance indicators, with the main goal being to improve patient outcomes. Newhouse [25, p.203] cautions however that “payment on specific process measures of quality […] can distort resource allocation to the measured areas and away from unmeasured areas”. Hence, a disproportionate focus on measured aspects can be detrimental to aspects of care that are not incentivized [26]. Via P4C, a designated provider receives a payment to coordinate patient care across a set of services. This is intended to provide financial leeway for patient–provider and provider–provider communication, and to limit unnecessary services, and may furthermore increase “flexibility in how, where, and by whom care is provided” [12, p.5].

### Network Incentives

Theoretically, all payment models in the taxonomy can provide incentives at the network level. Group-level or network-level payments or ‘rewards’ stimulate structural changes that are seen as preconditions for optimized patient care [11]. A switch from provider-level to network-level reimbursement implies a switch from individual (i.e., provider or organizational) incentives to network incentives. The terms network and groups are used interchangeably in the literature on monetary incentives that underpin payment models. In general, network-level incentives seem to be most effective when the delivery of health care services encompasses “significant interdependencies between group members” [27]. This presumes that, between network providers, high levels of clinical, professional, and organizational integration are present [28]. The intensity of network incentives might be attenuated by an increase in the number of providers working under the same target [29]. That is, an increase in network size leads to a weakening of incentives. Similarly, evidence from systematic reviews indicates that individual-level rewards are more powerful than network-level or group-level rewards [21]. In addition to the properties of the specific payment models discussed in the previous paragraph, such idiosyncrasies of network incentives might also influence performance.

## Research Methods

Given the broad nature of the research question [30], the polysemous nature of networks in health care, and the lack of uniform terminology of payment models [10], a scoping review was conducted. Scoping reviews are appropriate for topics where the field of literature is large, complex, ambiguous, and lacking in conceptual boundaries [31]. In our review, we complied with PRISMA-ScR reporting guidelines [32] and followed the five steps specified in the York framework, thereby allowing an iterative process. The process framework consists of (i) identifying the research question (see Introduction), (ii) identifying relevant studies, (iii) study selection, (iv) data charting, and (v) reporting on results [33]. In order to assess the evidence quality of studies, the Effective Practice and Organization of Care (EPOC) criteria table was adapted from Minkman et al. [34]. Evidence levels range from A (systematic reviews and RCTs), through B (controlled studies) and C (non-controlled studies), to D (descriptive, non-analytical studies).

### Identifying Relevant Studies

To identify relevant studies, a broad systematic search was conducted in six bibliographical databases. An information specialist with expertise in improving literature retrieval for systematic reviews [see 35] was consulted to draft the search strings. The initial string consisted of terms similar to ‘payment model’ and ‘interorganizational network’. A first search of four databases (Embase, Medline Ovid, Cochrane Central Register of Controlled Trials, and Web of Science Core Collection) yielded 3892 hits. Author 1 perused a sample of the identified studies to gain familiarity with concepts and identify additional terms that could serve as input for refining the search string [30]. This modified string was used for the second search in October 2019 and yielded 6069 hits including duplicates. For this search, two additional databases were consulted (EconLit ProQuest and CINAHL EBSCOhost) to further broaden the scope. The literature search was updated in November 2021, eventually yielding a total of 6953 studies including duplicates. Studies up to that date have been included with no earliest cut-off date set. Both the initial and final search strings are presented in the supplementary file. Alongside this bibliographical database search, reference lists were consulted to identify further studies that were eligible for inclusion.

### Study Selection

**Figure 1:**
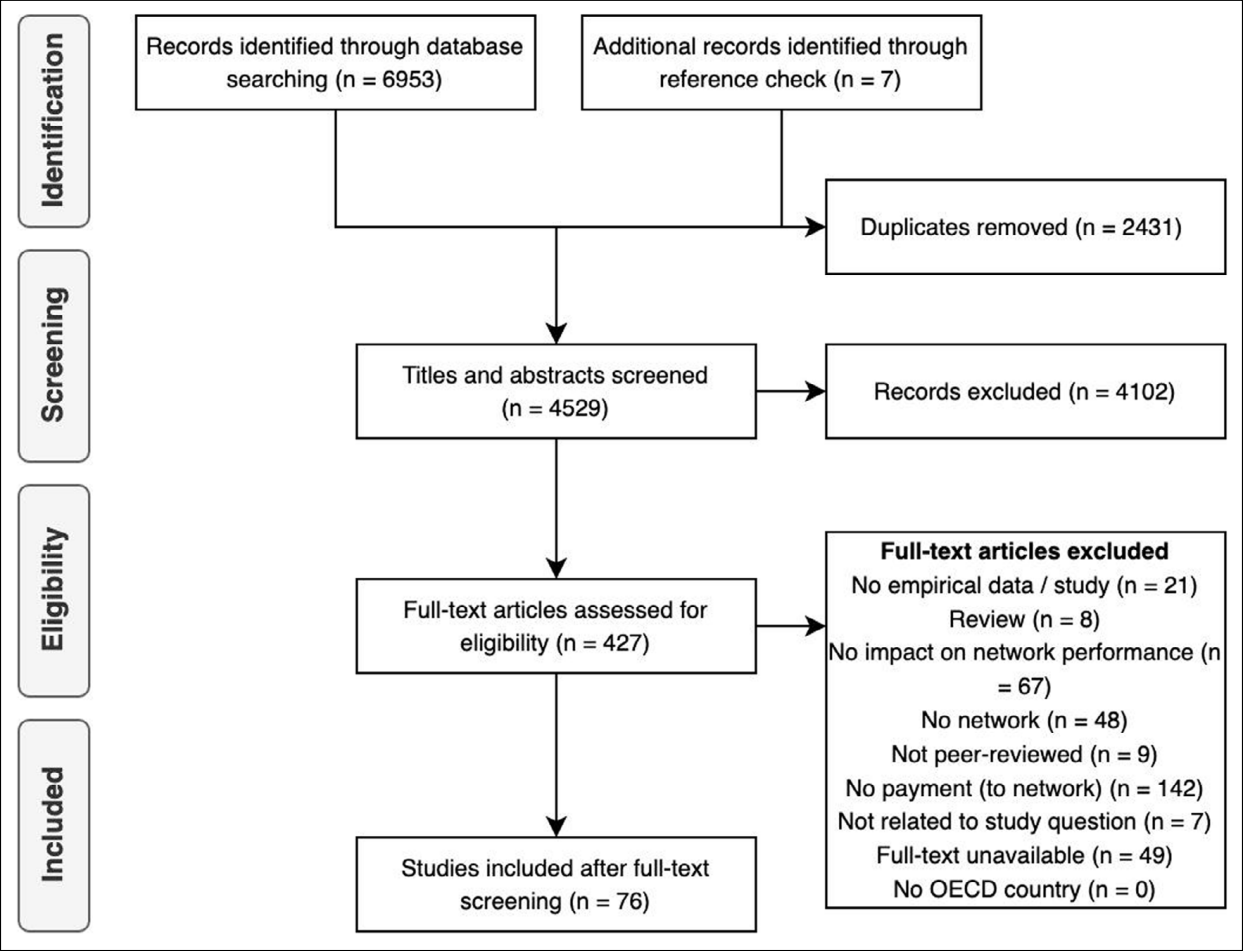
Flow diagram of screening process.

Studies were included if they were of an empirical nature, peer-reviewed, reported an impact on network performance, described a network-level payment model intervention, and were from an OECD country. OECD countries were chosen since the social and health challenges in these countries call for a well-coordinated system approach [36] that networks can contribute to. Systematic reviews were excluded (although their reference lists were scanned for studies eligible for inclusion) as well as articles where the full text could not be retrieved and where the contents were evidently not related to our research question. A concise list of the exclusion criteria can be found in ***Figure 1***, in which the screening process following the PRISMA guidelines is also illustrated [37]. All potential abstracts and titles were imported into EndNote X9 [38]. After deduplication, the remaining titles and abstracts were exported to an MS Excel workbook for further manual screening. All four authors were involved in the process. Before actual screening began, a sample of 90 papers was discussed to align the team members’ interpretations of the exclusion criteria. For each potential inclusion, title and abstract screening was conducted by at least two reviewers independently in a double-blind fashion. Author 1 screened all titles and abstracts, and Author 2, 3, and 4 each screened one-third of the total. Inconsistencies were resolved between the two reviewers who had screened the specific title and abstract. Once this filtering process was completed, the full texts of the still potentially relevant papers were screened by Author 1, and another reviewer was consulted if there were doubts as to whether to include an article.

### Data Charting

First, each study was analysed to identify its year, author, country, methodology, intervention program, network configuration, payment model, payment flow, study population, sample size, the investigated indicators of performance, and if the performance on each indicator increased (+), decreased (–) or if there was no (statistically significant) effect (0) under the use of the payment model. The taxonomy discussed in the theoretical framework section was used to code payment models. A distinction is made between payment flows from payer-to-network (i.e., to the network) and network-to-provider (i.e., in the network). As a final step, all the indicators were inductively placed in one of four categories [39]: (i) quality of care, (ii) utilization, (iii) spending, and (iv) other consequences. The fourth category is used for indicators that cannot be assigned to any of the first three categories. These tend to be more abstract measures such as ‘level of collaboration’ or ‘level of integration’. A narrative synthesis of the evidence was conducted.

## Results

In total, 6960 studies were identified, including seven additional studies that were identified through reference list checks (see ***Figure 1***). Of those, 427 were found eligible for full-text screening. This screening eventually reduced the number of studies to include in the qualitative synthesis to 76.

### Study Characteristics

A comprehensive overview of all the included studies can be found in ***Table 4*** (see below). Most articles stem from the most recent decade *(N =* 71), and only two of the older five studies were published before 2000. Studies mainly employed quantitative research designs, and, if not, mixed-method designs were employed (see ***Table 2***). Most studies were performed in the USA (*N* = 70), the others coming from Germany (*N* = 2) and the Netherlands (*N* = 4). This might explain the dominance of payments to ACOs as the networks under investigation. Capitation-based payments (*N =* 4), disease-based bundled payments (*N* = 5) and P4Ps (*N =* 8) were addressed in a total of 17 studies, while the remaining studies focused on global payments. The latter were often combined with additional components such as shared savings (*N =* 45), shared savings plus P4P (*N =* 13), and pay-for-coordination (*N =* 1). Most studies lacked precise network configuration descriptions and payment flows to a network (N = 68) were far more common than payment flows in a network (N = 8). The studied populations ranged from disease-specific groups to entire populations served by a network. The quality of evidence was mixed, but consisted predominantly of controlled studies (N = 65) (see ***Table 3***). For studies with evidence level B, the results presented in ***Table 4*** are statistically significant. For evidence C level studies, significance was only reported in two studies [40,41]. Given the exclusion criteria we had set, no studies were graded A (RCTs) or D (descriptive studies).

**Table 2:** Summary of empirical research.


COUNTRY	MAIN PAYMENT MODEL	RESEARCH DESIGN	PAYMENT FLOW

United States (*N* = 70)	Capitation (*N =* 4)	Quantitative (*N* = 66)	To network (*N =* 68)

Netherlands (*N* = 4)	Disease-based bundled payment (*N* = 5)	Mixed (*N* = 10)	In network (*N* = 8)

Germany (*N* = 2)	P4P (*N* = 8)		

	Global payment (*N* = 59)		


**Table 3:** Evidence quality of included studies.


LEVEL	DESCRIPTION	#

**A1**	**Systematic review**	
	Review of data from multiple RCT studies	0

**A2**	**Randomized trial**	
	Comparative study with (random) intervention and control group design	0

**B**	**Controlled study**	
	Trial with intervention and control group and comparisons on outcome	
	B1 Multiple measurement points	60
	B2 One measurement point	5

**C**	**Non-controlled study**	
	C1 Multiple case, multiple measurement points	4
	C2 Multiple case, one measurement point	1
	C3 Single case, multiple measurement points	4
	C4 Single case, one measurement point	2

**D**	**Descriptive, non-analytical**	0
	D1 Multiple projects	0
	D2 Single project	0
	D3 Literature review	0


**Table 4:** Summary of included articles. Abbreviations: HMO, Health Maintenance Organization; GP, general practitioner; ED, emergency department; HbA1C, average blood glucose levels for last two to three months; LDL(-C), low-density lipoprotein (cholesterol); SUD, substance use disorder; P4P, Pay-for-performance; SNF, skilled nursing facility; AMI, acute myocardial infarction; COPD, chronic obstructive pulmonary disease; CHF, congestive heart failure; ICU, intensive care unit; IRF, inpatient rehabilitation facility; HHA, home health agency; LTC, long-term care; AAA, abdominal aortic aneurysm; CABG, coronary artery bypass grafting; ACO, Accountable Care Organization; FFS, fee-for-service; DMP, disease management program. SBI; Screening and Brief Intervention (for SUD); PDC, proportion of days covered; ESRD, end stage renal disease; AVR, aortic valve replacement; MSSP, Medicare Shared Savings Program; AQC, Alternative Quality Contract; PGPD, Physician Group Practice Demonstration.


REFERENCE #	YEAR	FIRST AUTHOR	METHOD	COUNTRY	PROGRAM	NETWORK CONFIGURATION	PAYMENT MODEL(S)	FLOW	STUDY POPULATION	INTERVENTION *N*	CONTROL *N*	INDICATOR	QUALITY OF CARE	UTILIZATION	SPENDING	OTHER	EPOC

42	1995	Schlenker	QN	USA	HMO	Not specified	Capitation	To	Medicare home health beneficiaries	181 patients	1,079 patients	Patient discharge		+			C2

												Home health visits		+			

												Cost per patient			+		

												Episode length		+			

43	1995	Robinson	Mixed	USA	HMO	Integrated physician medical groups in California	Capitation	To	Capitated HMO enrollees in six integrated physician medical groups	Not reported	Not reported	Physician visits		–			C1

44	2014	Marton	QN	USA	Passport (P) and Kentucky Health Select Plan (K)	Primary care practices in regional managed care networks	Capitation + P4P (P) P4P (K)	In	Children enrolled in Medicaid	1,890 patients (P) and 4,273 patients (K)	2,816 patients (P) and 9,317 patients (K)	Outpatient utilization (P/K)		+/+			B1

												Professional utilization (P/K)		+/–			

45	2017	Mandal	QN	USA	HMO	Provider group with 7 clinic locations and 25 primary care specialists	Capitation + risk-and-gain sharing	To	Community-dwelling Medicare Advantage enrollees ≥65 years	1,230 patients	1,230 patients	Office-based visits		+			B1

												ED hospital visits		+			

												Inpatient hospital admissions		+			

												30-day readmission	0				

												60-day readmission	0				

												Preventive visits		+			

												Screening mammography		+			

												Screening colonoscopy		0			

48	2011	De Bakker	Mixed	NL	DMP for diabetes	Care groups, consisting of health care providers such as GPs, laboratories, dietitians and medical specialists	Disease-based bundled payment	To	Diabetes patients assigned to care group	Not reported (10 care groups)	Not reported	Collaboration				+	C4

												Process quality				+	

												Transparency				+	

												GP domination of care groups				–	

												Administrative burden				–	

												Price variations				–	

47	2014	Busse	Mixed	NL	DMP for diabetes	Care groups, consisting of health care providers such as GPs, laboratories, dietitians and medical specialists	Disease-based bundled payment	To	Diabetes patients assigned to care group	Not reported	Not reported	Specialist care use		+			B1

												Control of blood pressure and cholesterol		+			

												HbA1C		+			

												Regular check-ups		+			

												Foot exams		+			

												Kidney exams		+			

												Eye testing		–			

												Total medical spending			–		

46	2015	Mohnen	QN	NL	DMP for diabetes	Care groups, consisting of health care providers such as GPs, laboratories, dietitians and medical specialists	Disease-based bundled payment	To	Diabetes patients assigned to care group	20,257 patients	43,754 patients	Curative health care spending			–		B1

49	2021	Karimi	QN	NL	DMP for diabetes, COPD and vascularrisk management (VRM)	Care groups, consisting of health care providers such as GPs, laboratories, dietitians and medical specialists	Disease-based bundled payment	To	Patients enrolled in a bundled payment for diabetes, COPD, or increased vascular risk	807,197 patients (diabetes), 1,039,406 (VRM), 267,843 (COPD)	988,480 patients	Diabetes total cost			–		B1

												Diabetes medical specialist cost			–		

												Diabetes primary care cost			+		

												Diabetes medication cost			–		

												Diabetes bundled payment cost			–		

												VRM total cost			–		

												VRM medical specialist cost			–		

												VRM primary care cost			+		

												VRM medication cost			–		

												VRM bundled payment cost			–		

												COPD total cost			–		

												COPD medical specialist cost			–		

												COPD primary care cost			+		

												COPD medication cost			–		

												COPD bundled payment cost			–		

50	2021	Navathe	QN	USA	MSSP	Not specified	Disease-based bundled payment	In	Medicare fee-for-service beneficiaries	24,884 patients	70,208 patients	Post discharge institutional spending medical episode (non-ACO/ACO)			+/+		B1

												Medical episode mortality (non-ACO/ACO)	0/0				

												Medical episode 90-day readmissions (non-ACO/ACO)	0/+				

												Medical episode discharge to SNF/IRF (non-ACO/ACO)		0/0			

												Medical episode discharge to HH (non-ACO/ACO)		+/+			

												Medical episode length of stay SNF (non-ACO/ACO)		+/+			

												Post discharge institutional spending surgical episode (non-ACO/ACO)			+/+		

												Surgical episode mortality (non-ACO/ACO)	0/0				

												Surgical episode 90-day readmissions (non-ACO/ACO)	+/+				

												Surgical episode discharge to SNF/IRF (non-ACO/ACO)		+/+			

												Surgical episode discharge to HH (non-ACO/ACO)		+/+			

												Surgical episode length of stay SNF (non-ACO/ACO)		+/+			

52	2007	Mandel	QN	USA	Not specified	Physician-hospital organization consisting of primary care practices (PCPs)	P4P	To/in	Children with asthma	13,380 patients in 44 PCPs	Not reported	Perfect care delivery	+				C3

												Influenza vaccination rates	+				

51	2006	Levin-Scherz	QN	USA	Partners Community HealthCare (PCHI)	Network composedof 15 regional service organizations	P4P	To	PCHI patients	Variedper measure	Variedper measure	HbA1C screening		+			B1

												Diabetic LDL screening		+			

												Nephropathy screening		+			

												Diabetic eye exams		+			

												Paediatric asthma controller use		0			

53	2010	Atkinson	QN	USA	Long Island Health Network	Clinically integrated network of 10 hospital facilities	P4P	In	Not specified	Not reported	Not reported	Quality (overall composite measure)	+				C3

												Hospital average length of stay		+			

54	2018	Rieckmann	Mixed	USA	Coordinated Care Organization	Integrated financing and service delivery for medical, behavioural, and dental health	P4P	In	Members enrolled in CCO	Not specified	Not specified	SUD screening		+			C1

												SUD treatment initiation		–			

												SUD treatment engagement		–			

55	2015	Hibbard	Mixed	USA	Fairview Pioneer ACO	PCPs working in a Pioneer accountable care organization	P4P	In	Fairview PCPs	85 respondents	Not applicable	Efforts into increasing patient activation and patient self-management				0	C3

												Becoming more patient-centred				0	

56	2016	Gleeson	QN	USA	Partner for Kids	Physicians in a paediatric accountable care organization	P4P	In	Community physicians who received P4P incentives	203 physicians across 50 practices	2763 physicians across 82 practices	Adolescent well care visits		+			B1

												Well child visits at 3–6 years^1^		+/0			

												Asthma at 12–18 years	0/0				

												Asthma at 5–11 years	0/0				

												Immunizations (adolescents)	0/–				

												Meningococcal immunizations (adolescents)	0/–				

												Td/Tdap immunizations (adolescents)	0/–				

												Immunizations (children)	0/–				

												DTP immunizations (children)	+/–				

												Hepatitis A immunizations (children)	0/–				

												IPV immunizations (children)	+/–				

												MMR immunizations (children)	0/0				

												Pneumococcal conjugate immunizations (children)	+/0				

												Varicella immunizations (children)	0/–				

												Pharyngitis	0/0				

												Upper respiratory infection	0/0				

												ADHD maintenance		0/0			

												ADHD initiation		0/0			

												Lead screening		+/0			

												Influenza	0/0				

												Rotavirus	+/–				

57	2020	Ganguli	QN	USA	MSSP	Not specified	P4P	In	ACOs using cost reduction-based specialist compensation (P4P)	41 ACOs	119 ACOs	Shared savings			0		B2

												Outpatient spending			0		

												Specialist visits		0			

115	2017	Afendulis	Mixed	USA	Total Cost and Care Improvement (TCCI)	Primary care physician panels, consisting of at least 5-15 physicians and 1000 patients	Global payment + shared savings + pay-for-coordination	To	CareFirst BlueCross BlueShield Total Care and Cost Improvement Program enrollees	298,463 patients	537,778 patients	Outpatient spending			0		B1

												Specialist visits		0			

												Primary care visits		0			

												Inpatient spending			0		

												Outpatient spending			0		

												Total spending			0		

103	2017	Stuart	QN	USA	AQC	Not reported	Global payment + P4P + shared savings	To	BCBSMA HMO and POS (point of service) plan enrollees	10,817 patients	50,576 patients	SUD service utilization 2		0/+			B1

												SUD spending			0/0		

												SUD identification		0/+			

												SUD initiation		0/+			

												SUD engagement		0/0			

106	2013	McWilliams	QN	USA	AQC	Not reported	Global payment + P4P + shared savings	To	Elderly FFS Medicare beneficiaries in Massachusetts treated by AQC-affiliated providers	417,182 person-years	1,344,143 person-years	Total spending			+		B1

												Admission rate for ambulatory care–sensitive conditions related to cardiovascular disease or diabetes		0			

												30-day readmission	0				

												Mammography screening		0			

												LDL-C testing (diabetes and cardiovascular)		+			

												HbA1C testing		0			

												Diabetes retinal examination		0			

107	2011	Song	QN	USA	AQC	Not reported	Global payment + P4P + shared savings	To	Enrollees whose PCPs were in the AQC system	380,142 enrollees	1,351,446 enrollees	Medical spending			+		B1

												Paediatric care quality	+				

												Adult preventive care quality	0				

												Chronic care management quality	+				

108	2012	Song	QN	USA	AQC	Not reported	Global payment + P4P + shared savings	To	BCBSMA enrollees	612,547 enrollees	1,339,798 enrollees	Medical spending			+		B1

												Paediatric care quality	+				

												Adult preventive care quality	+				

												Chronic care management quality	+				

109	2014	Song	QN	USA	AQC	Not reported	Global payment + P4P + shared savings	To	Persons in four cohorts of AQC organizations, defined by first contract year: 2009, 2010, 2011, 2012	1,348,235 enrollees	966,813 enrollees	Medical spending			+		B1

												Chronic disease management quality	+				

												Adult preventive care quality	+				

												Paediatric care quality	+				

104	2016	Huskamp	QN	USA	AQC	Not reported	Global payment + P4P + shared savings	To	Adults between 18–64 years enrolled in BCBSMA HMO or POS (point of service) plans	533,568 person-years	2,999,221 person-years	Any tobacco cessation treatment use		+			B1

												Varenicline or bupropion use		+			

												Nicotine replacement therapy use		0			

												Tobacco cessation counselling visit use		+			

												Combination therapy (pharmacotherapy plus counselling) use		+			

												≥90-day supply of tobacco cessation		+			

112	2014	Afendulis	QN	USA	AQC	Not reported	Global payment + P4P + shared savings	To	BCBSMA HMO and POS (point of service) plan enrollees	332,624 enrollees	1,296,399 enrollees	Drug utilization		0			B1

												Drug spending			0		

113	2018	Donohue	QN	USA	AQC	Not reported	Global payment + P4P + shared savings	To	Individuals with alcohol use disorders (AUD) and/or opioid use disorders (OUD)	8,956 person-years	40,884 person-years	Medication treatment use		0			B1

110	2014	Chien	QN	USA	AQC	Not reported	Global payment + P4P + shared savings	To	BCBSMA HMO enrollees 0-21 years with and without special health care needs (CSHCN)	126,975 enrollees	415,331 enrollees	Quality measures tied to P4P	+				B1

												Quality measures not tied to P4P	0				

												Medical spending			0		

111	2017	Pimperl	QN	DE	Gesundes Kinzigtal	Not reported	Global payment + P4P + shared savings	To	Gesundes Kinzigtal enrollees	Varied per measure	Varied per measure	Mortality rate	0				B1

												Average age at time of death	+				

												Years of potential life lost	+				

105	2012	Hildebrandt	QN	DE	Gesundes Kinzigtal	Population-wide integrated care system that covers all sectors and indications of care with a group of providers	Global payment + P4P + shared savings	To	Gesundes Kinzigtal enrollees	Varied per measure	Varied per measure	Hospitalization		+			B1

												Medical spending			+		

114	2017	Blewett	Mixed	USA	Integrated Health Partnership Minnesota	Integrated health partnerships deliver the full scope of primary care services, and coordinate access to specialty providers and hospitals	Global payment + P4P + shared savings	To	Minnesota Health Care Program enrollees	Not reported	Not reported	Forging of community partnerships				+	C4

												Service integration				+	

58	2014	Sandberg	Mixed	USA	Hennepin Health	Hennepin County Human Services and Public Health Department; Hennepin County Medical Center, NorthPoint Health and Wellness Center, Metropolitan Health Plan (HMO), all covering physical, behavioural and social services.	Global payment + shared savings	To	Adults without dependent children	Not reported	Not reported	ED visits		+			C3

												Outpatient visits		+			

												Hospitalization		0			

												Patients receiving optimal diabetes, vascular and asthma care	+				

41	2017	Narayan	QN	USA	MSSP	Not reported	Global payment + shared savings	To	Medicare beneficiaries in ACOs	5,329,831 beneficiaries	Not reported	Mammography screening use		+			C1

40	2018	Fraze	QN	USA	MSSP	Not reported	Global payment + shared savings	To	MSSP ACOs	162 ACOs	Not reported	All-or-nothing diabetes composite	+				C1

												HbA1C controlled	+				

												LDL controlled	+				

												Blood pressure <140/90	+				

												Tobacco non-use	+				

												Aspirin use	+				

59	2014	Pope	Mixed	USA	PGPD	Not reported	Global payment + shared savings	To	Beneficiaries assigned to PGPs	1,776,387 person-years	1,579,080 person-years	Medical spending			+		B1

												Hospitalizations		+			

												ED visits		+			

												HbA1C testing		+			

												LDL-C testing		+			

												Medical attention for nephropathy	+				

												Diabetes eye exam		+			

												Left ventricular ejection fraction testing		0			

												Lipid profile		+			

												Breast cancer screening		+			

90	2019	Kim	QN	USA	MSSP	Not reported	Global payment + shared savings	To	Medicare FFS beneficiaries with a cancer diagnosis who were 66 years or older and died in 2013-2014	9,033 beneficiaries	9,033 beneficiaries	≥1 ICU admission (Aggressive end-of-life care)		–			B2

												≥2 Hospitalizations (Aggressive end-of-life care)		+			

												≥2 ED visits (Aggressive end-of-life care)		0			

												Chemotherapy ≤2 weeks (Aggressive end-of-life care)		0			

												No hospice or enrolment ≤3 days (Aggressive end-of-life care)		0			

91	2014	Colla	QN	USA	PGPD	Not reported	Global payment + shared savings	To	FFS Medicare patients assigned to PGPs	819,779 patients	934,621 patients	Discretionary carotid imaging use		0			B1

												Discretionary coronary imaging use		0			

												Discretionary carotid procedures use		0			

												Discretionary coronary procedures use		0			

												Non-discretionary carotid procedures use		0			

												Non-discretionary coronary procedures use		0			

60	2019	Rutledge	Mixed	USA	Medicaid ACO	Not reported	Global payment + P4P + shared savings	To	ACOs in Maine, Minnesota and Vermont	3 ACOs	Not specified	Primary care provider visits (Maine, Minnesota, Vermont)		–/–/0			B1

												Acute inpatient hospitalizations (Maine, Minnesota, Vermont)		+/–/+			

												ED visits (Maine, Minnesota, Vermont)		+/+/+			

												30-day readmissions (Maine, Minnesota, Vermont)	0/+/0				

												HbA1C testing (Maine, Minnesota)		0/+			

												Medication adherence for depression (Maine, Minnesota)	0/–				

												Developmental screening (Vermont)		+			

												Total spending (Maine, Minnesota, Vermont)			0/0/+		

												Inpatient spending (Maine, Minnesota, Vermont)			0/0/+		

												Professional spending (Maine, Minnesota, Vermont)			0/+/+		

												Pharmaceutical spending (Maine, Vermont)			0/+		

61	2019	Borza	QN	USA	MSSP	Not reported	Global payment + shared savings	To	Patients undergoing common surgical procedures at ACO-affiliated hospitals	80,501 patients	348,774 patients	Overall 30-day readmission	+				B1

												Readmission after AAA repair	0				

												Readmission after colectomy	0				

												Readmission after cystectomy	0				

												Readmission after Prostatectomy	0				

												Readmission after lung resection	0				

												Readmission after total knee arthroplasty	+				

												Readmission after total hip arthroplasty	0				

101	2019	Diana	QN	USA	Pioneer, MSSP	Not reported	Global payment + shared savings	To	ACO-affiliated hospitals	615 hospitals	2,847 hospitals	Communication with nurses (patient experience) (Pioneer/MSSP)				+/0	B2

												Communication with doctors (patient experience) (Pioneer/MSSP)				+/0	

												Responsiveness of hospital staff (patient experience) (Pioneer/MSSP)				0/0	

												Pain management (patient experience) (Pioneer/MSSP)				0/0	

												Communication about medications (patient experience) (Pioneer/MSSP)				0/0	

												Cleanliness of hospital environment (patient experience) (Pioneer/MSSP)				0/0	

												Quietness of hospital environment (patient experience) (Pioneer/MSSP)				0/0	

												Discharge information (patient experience) (Pioneer/MSSP)				0/0	

												Overall hospital rating (patient experience) (Pioneer/MSSP)				0/0	

												Recommend the hospital (patient experience) (Pioneer/MSSP)				0/0	

92	2019	Trinh	QN	USA	MSSP	Not reported	Global payment + shared savings	To	FFS, non-HMO beneficiaries	51,980 beneficiaries	222,800 beneficiaries	Rates of prostate specific antigen screening		0			B1

												Rates of prostate biopsy		0			

83	2017	Zhang	QN	USA	Pioneer	Not reported	Global payment + shared savings	To	FFS Medicare beneficiaries	316,366 beneficiaries	559,241 beneficiaries	Medicare Part D drug spending			0		B1

												Total prescriptions filled		0			

												Medicare Part A/B medical spending			+		

62	2017	Winblad	QN	USA	Pioneer, MSSP	Not reported	Global payment + shared savings	To	ACO-affiliated hospitals	226 hospitals	1,844 hospitals	30-day overall adjusted rehospitalization rate (MSSP/Pioneer)	+/+				B1

63	2019	Kaufman	QN	USA	MSSP	Not reported	Global payment + shared savings	To	MSSP hospitals	273 hospitals	1,490 hospitals	Discharge to home		–			B1

												30-day all-cause readmissions	0				

												Hospital length of stay		0			

												Days in the community	0				

												Mortality	0				

												Recurrent stroke within 1 year of hospitalization	0				

64	2019	Bain	QN	USA	MSSP	Not reported	Global payment + shared savings	To	MSSP hospitals	233 hospitals	3,100 hospitals	Probability of discharge to one-star (low-rated) SNFs	0				B1

												Probability of discharge to five-star (high-rated) SNFs	+				

93	2018	Resnick	QN	USA	MSSP	Not reported	Global payment + shared savings	To	Medicare Part A and B beneficiaries > 65 years	4,989,210 beneficiary-years	12,263,135 beneficiary-years	Breast cancer screening use		–			B1

												Colorectal cancer screening use		+			

												Prostate cancer screening use		+			

65	2018	Kim	QN	USA	MSSP	Not reported	Global payment + shared savings	To	MSSP hospitals	Varied per measure	Varied per measure	30-day hospital-wide all cause readmission rates	+				B1

												30-day readmissions rates for AMI	0				

												30-day readmissions rates for heart failure	+				

												30-day readmissions rates for pneumonia	+				

77	2019	Cole	QN	USA	MSSP	Not reported	Global payment + shared savings	To	Medicare Part A and B beneficiaries ≥ 67 years with prostate cancer	3,297 beneficiaries	24,088 beneficiaries	Radical prostatectomy spending			0		B1

												Radiation therapy (EBRT, IMRT, Brachytherapy) spending			0		

												Expectant management (no surgery, radiation treatment within the first 180 days after diagnosis) spending			0		

66	2016	Busch	QN	USA	Pioneer, MSSP	Not reported	Global payment + shared savings	To	Medicare beneficiaries ≥ 18 years with mental health illness	Not specified	Not specified	All mental health care spending (Pioneer 2012 performance year/Pioneer 2013 performance year/MSSP 2012 entry cohort/MSSP 2013 entry cohort)			+/0/0/0		B1

												Outpatient mental health care spending (Pioneer 2012 performance year/Pioneer 2013 performance year/MSSP 2012 entry cohort/MSSP 2013 entry cohort)			0/0/0/0		

												ED visits with mental health diagnosis spending (Pioneer 2012 performance year/Pioneer 2013 performance year/MSSP 2012 entry cohort/MSSP 2013 entry cohort)			+/0/0/0		

												Inpatient admissions with mental health diagnosis spending (Pioneer 2012 performance year/Pioneer 2013 performance year/MSSP 2012 entry cohort/MSSP 2013 entry cohort)			+/0/0/0		

												30-day mental health readmissions (Pioneer 2012 performance year/Pioneer 2013 performance year/MSSP 2012 entry cohort/MSSP 2013 entry cohort)	0/0/0/0				

												Outpatient mental health follow-up within 7 days of discharge (Pioneer 2012 performance year/Pioneer 2013 performance year/MSSP 2012 entry cohort/MSSP 2013 entry cohort)	0/0/0/0				

												Identified as having a depressive disorder (Pioneer 2012 performance year/Pioneer 2013 performance year/MSSP 2012 entry cohort/MSSP 2013 entry cohort)	0/0/–/0				

67	2015	McWilliams	QN	USA	Pioneer	Not reported	Global payment + shared savings	To	Fee-for-service Medicare beneficiaries	768,054 beneficiary-years	19,152,460 beneficiary-years	Total spending			+		B1

												30-day readmissions	0				

												Hospitalizations for ambulatory-care sensitive conditions		0			

												CHF hospitalizations		0			

												COPD or asthma hospitalizations		0			

												Cardiovascular disease or diabetes hospitalizations		0			

												Screening mammography (for women 65–69 years)		0			

												HbA1C testing		+			

												LDL-C testing		+			

												Diabetic retinal examination		+			

68	2016	McWilliams	QN	USA	MSSP	Not reported	Global payment + shared savings	To	Fee-for-service Medicare beneficiaries	884,810 (2012 cohort) and 1,015,722 beneficiary-years (2013 cohort)	10,924,440 (2012 cohort) and 14,587,259 beneficiary-years (2013 cohort)	Total spending (2012/2013 entry cohort)			+/0		B1

												30-day readmissions	0				

												Hospitalizations for ambulatory-care sensitive conditions		0			

												CHF hospitalizations		0			

												COPD or asthma hospitalizations (2012/2013 entry cohort)		+/0			

												Cardiovascular disease or diabetes hospitalizations		0			

												Screening mammography (for women 65–69 years)		0			

												HbA1C testing		0			

												LDL-C testing (2012/2013 entry cohort)		+/0			

												Diabetic retinal examination (2012/2013 entry cohort)		0/+			

												Low-value services provided		0			

79	2018	Lam	QN	USA	MSSP	Not reported	Global payment + shared savings	To	Medicare FFS beneficiaries ≥ 65 years with cancer	388,784 patients	233,296 patients	Lung cancer spending			0		B1

												Hematologic cancer spending			0		

												Gastrointestinal cancer spending			0		

												Breast cancer spending			0		

												Genitourinary cancer spending			0		

												Gynaecologic cancer spending			0		

												Head and neck cancer spending			0		

												Sarcoma spending			0		

												Melanoma spending			0		

												Central nervous system cancer spending			0		

												Metastatic disease (primary unknown) spending			0		

												Total spending			0		

												Inpatient spending			0		

												Outpatient cancer spending			0		

												Physician services spending			0		

												SNF spending			0		

												HHA spending			0		

												Hospice spending			0		

												Radiation therapy spending			0		

												Chemotherapy spending			0		

69	2018	Duggal	QN	USA	Pioneer, MSSP	Not reported	Global payment + shared savings	To	ACO-affiliated hospitals	129 Pioneer-affiliated hospitals and 342 MSSP-affiliated hospitals	3,907 hospitals	Heart failure 30-day readmission rate (MSSP/Pioneer)	+/0				B1

												AMI 30-day readmission rate (MSSP/Pioneer)	0/0				

												Pneumonia 30-day readmission rate (MSSP/Pioneer)	0/0				

81	2018	McWilliams	QN	USA	MSSP	Physician-group ACOs (narrow scope of provided services) and hospital-integrated ACOs (wider scope of provided services)	Global payment + shared savings	To	Fee-for-service Medicare beneficiaries	Hospital-integrated ACOs (132) and physician-group ACOs (203)	Not specified	Physician group ACO spending (2012/13/14 entry cohort)			+/+/+		B1

												Hospital-integrated ACO spending (2012/13/14 entry cohort)			+/0/0		

94	2019	Modi	QN	USA	MSSP	Not reported	Global payment + shared savings	To	Medicare part A and B FFS beneficiaries ≥ 66 years undergoing meniscectomy, vertebroplasty or hip fracture procedure	21,486 meniscectomy, 12,521 vertebroplasty and 13,930 hip fracture patients	54,770 meniscectomy, 32,018 vertebroplasty and 36,830 hip fracture patients	Arthroscopic partial meniscectomy (low-value procedure) use		0			B1

												Vertebroplasty (low-value procedure) use		0			

70	2016	Herrel	QN	USA	MSSP	Not reported	Global payment + shared savings	To	Patients aged 66 to 99 years that underwent major cancer surgery for nine solid organ cancers	19,439 patients	365,080 patients	30-day mortality	0				B1

												30-day readmissions	0				

												30-day major complications	0				

												Hospital length of stay	0				

76	2018	Borza	QN	USA	MSSP	Not reported	Global payment + shared savings	To	Medicare part A and B FFS beneficiaries ≥ 66 years with prostate cancer	5,065 patients	27,946 patients	Treatment rate in highest mortality risk (overtreatment)		+			B1

												Overall payments			0		

												Payments in highest mortality risk			0		

78	2016	Colla	QN	USA	Pioneer, MSSP	Not reported	Global payment + shared savings	To	(1) Medicare part A and B FFS beneficiaries and (2) Medicare part A and B FFS beneficiaries ≥ 66 years with multiple clinical conditions (clinically vulnerable)	Not specified	Not specified	Total spending (Pioneer 2012 entry cohort/MSSP 2012 entry cohort/MSSP 2013 entry cohort)			+/+/+		B1

												Spending among clinically vulnerable beneficiaries (Pioneer 2012 entry cohort/MSSP 2012 entry cohort/MSSP 2013 entry cohort)			+/+/+		

95	2018	Resnick	QN	USA	MSSP	Not reported	Global payment + shared savings	To	Medicare Part A and B beneficiaries > 65 years	13,460,798 person-years	40,010,199 person-years	Breast cancer screening use among appropriate candidates		+			B1

												Colorectal cancer screening use among appropriate candidates		+			

												Prostate cancer screening use among appropriate candidates		0			

82	2015	Schwartz	QN	USA	Pioneer	Not reported	Global payment + shared savings	To	Medicare Part A and B beneficiaries	693,218 person-years	17,453,423 person-years	Total low-value services use		+			B1

												Total low-value services spending			+		

												Cancer screening use		+			

												Testing use		+			

												Preoperative services use		0			

												Imaging use		+			

												Cardiovascular tests and procedures use		+			

												Other invasive procedures use		0			

												Higher-priced low-value services use		0			

												Lower-priced low-value services use		+			

												More patient sensitive low-value services use		+			

												Less patient sensitive low-value services use		+			

80	2017	McWilliams	QN	USA	MSSP	Not reported	Global payment + shared savings	To	Medicare Part A and B beneficiaries	Not specified	Not specified	SNF spending (2012/2013/2014 entry cohort)			+/+/0		B1

71	2019	Markovitz	QN	USA	MSSP	Not reported	Global payment + shared savings	To	Medicare FFS beneficiaries	835,100 beneficiaries	Not reported	Total spending			0		B1

												HbA1C testing (% meeting quality indicator)	0				

												LDL-C testing (% meeting quality indicator)	–				

												Diabetic retinal examination (% meeting quality indicator)	0				

												All 3 diabetes measures (% meeting quality indicator)	0				

												Mammography (% meeting quality indicator)	0				

96	2018	Barnett	QN	USA	MSSP	Not reported	Global payment + shared savings	To	Medicare FFS Part A and B beneficiaries	Not specified	Not specified	All specialist visits in primary care oriented ACOs (2012/2013/2014 entry cohort)		+/0/0			B1

												All specialist visits in specialty oriented ACOs (2012/2013/2014 entry cohort)		0/0/0			

												New specialist visits in primary care oriented ACOs (2012/2013/2014 entry cohort)		+/0/+			

												New specialist visits in specialty oriented ACOs (2012/2013/2014 entry cohort)		0/0/0			

97	2017	McWilliams	QN	USA	MSSP	Not reported	Global payment + shared savings	To	Medicare FFS Part A, B and D beneficiaries with cardiovascular disease or diabetes	Not specified	Not specified	Statin use (2012/2013/2014 entry cohort)		0/0/0			B1

												Statin PDC (2012/2013/2014 entry cohort)		0/0/0			

												ACE inhibitor/ARB use (2012/2013/2014 entry cohort)		0/0/0			

												ACE inhibitor/ARB PDC (2012/2013/2014 entry cohort)		0/0/0			

												β-Blockers use (2012/2013/2014 entry cohort)		0/0/0			

												β-Blockers PDC (2012/2013/2014 entry cohort)		+/0/0			

												Thiazide diuretics use (2012/2013/2014 entry cohort)		0/+/0			

												Thiazide diuretics PDC (2012/2013/2014 entry cohort)		0/0/0			

												Calcium channel blockers use (2012/2013/2014 entry cohort)		0/0/0			

												Calcium channel blockers PDC (2012/2013/2014 entry cohort)		0/0/0			

												Metformin use (2012/2013/2014 entry cohort)		0/0/0			

												Metformin PDC (2012/2013/2014 entry cohort)		+/+/0			

72	2015	Nyweide	QN	USA	Pioneer	Not reported	Global payment + shared savings	To	Medicare FFS beneficiaries	675,712 beneficiaries in 2012 and 806,258 beneficiaries in 2013	13,203,694 beneficiaries in 2012 and 12,134,154 beneficiaries in 2013	Total Medicare spending (2012/2013 performance year)			+/+		B1

												All inpatient hospital (Part A) spending (2012/2013 performance year)			+/+		

												Physician (Part B) spending (2012/2013 performance year)			+/+		

												Hospital outpatient spending (2012/2013 performance year)			+/0		

												SNF spending (2012/2013 performance year)			+/0		

												Home health spending (2012/2013 performance year)			+/0		

												Hospice spending (2012/2013 performance year)			+/0		

												Durable medical equipment spending (2012/2013 performance year)			+/+		

												Acute care inpatient days (2012/2013 performance year)		+/+			

												Inpatient admissions through ED (2012/2013 performance year)		+/+			

												IRF or LTC facility days (2012/2013 performance year)		0/+			

												All-cause 30-day readmissions (2012/2013 performance year)	0/0				

												Post discharge physician visits within 7 days (2012/2013 performance year)	+/+				

												Post discharge physician visits within 14 days (2012/2013 performance year)	0/+				

												Post discharge physician visits within 30 days (2012/2013 performance year)	0/0				

												Primary care evaluation and management visits (2012/2013 performance year)		+/+			

												Procedures use (2012/2013 performance year)		+/+			

												Imaging services use (2012/2013 performance year)		+/+			

												Tests use (2012/2013 performance year)		+/+			

												ED visits (2012/2013 performance year)		+/+			

												Observation stays (2012/2013 performance year)		0/–			

												SNF days (2012/2013 performance year)		+/0			

												Home health visits (2012/2013 performance year)		+/0			

												Hospice days (2012/2013 performance year)		+/0			

98	2018	Lin	QN	USA	MSSP	Not reported	Global payment + shared savings	To	ACO-affiliated rural health clinics (RHCs)	19 RHCs	484 RHCs	Risk-adjusted diabetes hospitalization rate		0			B2

73	2013	Colla	QN	USA	PGPD	Not reported	Global payment + shared savings	To	Medicare FFS beneficiaries with cancer	123,249 beneficiaries	865,532 beneficiaries	Acute care spending			+		B1

												Imaging spending			0		

												Deaths occurring in hospital	0				

84	2019	Lam	QN	USA	MSSP	Not specified	Global payment + shared savings	To	ACO cancer decedents	12,248 patients	12,248 patients	Total spending			0		B1

												Inpatient spending			0		

												Outpatient spending			0		

												Physician services spending			0		

												SNF spending			0		

												Home health spending			0		

												Hospice spending			0		

												Radiation therapy spending			0		

												Chemotherapy spending			0		

												≥1 Emergency room visits (180 days/30 days prior to death)		0/0			

												≥1 Inpatient hospitalizations (180 days/30 days prior to death)		–/0			

												≥1 ICU admission (180 days/30 days prior to death)		0/0			

85	2020	Bakre	QN	USA	MSSP	Not specified	Global payment + shared savings	To	Medicare fee-for-service beneficiaries on long-term dialysis	26,694 patients	167,817 patients	Total spending			+		B1

99	2021	Modi	QN	USA	MSSP	Not specified	Global payment + shared savings	To	ACO hospitals	707 hospitals	1,770 hospitals	AAA treatment rate		0			B1

												AVR treatment rate		0			

												Carotid endarterectomy/stent treatment rate		0			

												Colectomy treatment rate		0			

												Lung lobectomy treatment rate		0			

												Prostatectomy treatment rate		0			

												Proportion of AAA surgery using EVAR				0	

												Proportion of AVR using TAVR				0	

												Proportion of carotid surgery using stenting				0	

												Proportion of colectomy surgery using minimally invasive approach				0	

												Proportion of lobectomy surgery using minimally invasive approach				0	

												Proportion of prostatectomy using minimally invasive approach				0	

86	2021	Chang	QN	USA	MSSP	Not specified	Global payment + shared savings	To	Long-term nursing home Medicare fee-for-service beneficiaries	121,690 patients	121,690 patients	Evaluation & management visits		0			B2

												Proportion of evaluation & management visits to primary care physicians		+			

												Total admissions		+			

												ACSC admissions		+			

												30-day readmissions		0			

												Observation stays		0			

												ED visits		+			

												Total spending			0		

87	2021	Erfani	QN	USA	Medicare ACO	Not specified	Global payment + shared savings	To	Medicare fee-for-service beneficiaries aged 65 years or older with cancer	517,623 patients	348,909 patients	Lung cancer spending			0		B1

												Hematologic cancer spending			0		

												Gastrointestinal cancer spending			0		

												Breast cancer spending			0		

												Genitourinary cancer spending			0		

												Gynaecologic cancer spending			0		

												Head and neck cancer spending			0		

												Sarcoma spending			0		

												Melanoma spending			0		

												Central nervous system cancer spending			0		

												Metastatic disease (primary unknown) spending			0		

100	2021	Acevedo	QN	USA	MSSP	Not specified	Global payment + shared savings	To	Medicare beneficiaries	853,953 patients with disability (D) and 2,917, 299 patients aged 65 years or older^3^ (65)	1,675,928 and 5,492,387 patients	Any outpatient mental health visits (D/65)		+/–			B1

												Any outpatient substance use visits (D/65)		+/0			

												Any inpatient mental health stays (D/65)		+/+			

												Any inpatient substance use stays (D/65)		+/0			

												Number of inpatient mental health visits (D/65)		+/+			

												Number of inpatient substance use visits (D/65)		0/–			

												Adequate care for patients with depression (D/65)	–/–				

102	2020	Lee	QN	USA	MSSP	Not specified	Global payment + shared savings	To	Vulnerable ACO beneficiaries in physician group panels	1,024,833 patients	2,912,043 patients	Proportion of black patients				0	B1

												Proportion of patients that are dually enrolled in Medicare and Medicaid				0	

												Proportion of patients that live in areas with higher poverty rates				0	

												Proportion of patients that live in areas with higher unemployment rates				0	

89	2020	McWilliams	QN	USA	MSSP	Not specified	Global payment + shared savings	To	ACOs across different entry cohorts	114 ACOs (2012 entry cohort), 106 (2013), 115 (2014)	Not specified	Spending (2012 entry cohort) (2013/14/15)			0/+/+		B1

												Spending (2013 entry cohort) (2013/14/15)			0/+/+		

												Spending (2014 entry cohort) (2014/15)			0/0		

75	2019	Zhang	QN	USA	Commercial ACO	HMO, large independent practice association of physicians and hospital system	Global payment + shared savings	To	Enrolled members of commercial HMO	40,483 patients	20,275 patients	Inpatient and outpatient payments (2010/11/12/13/14)			–/–/0/0/0		

												PCP visits (2010/11/12/13/14)		0/0/0/–/–			

												Specialist visits (2010/11/12/13/14)		–/–/–/–/–			

												ED visits (2010/11/12/13/14)		0/0/–/0/0			

												Inpatient admissions (2010/11/12/13/14)		0/0/0/0/–			

												30-day readmissions (2010/11/12/13/14)	0/0/0/0/0				

												Breast cancer screening (2010/11/12/13/14)		0/0/0/+/+			

												Cervical cancer screening (2010/11/12/13/14)		0/0/0/+/+			

												Colorectal cancer screening (2010/11/12/13/14)		0/0/0/+/+			

												HPV vaccine (2010/11/12/13/14)	0/0/0/0/+				

												Immunizations (combination 1) (2010/11/12/13/14)	+/0/+/0/+				

												Meningococcal immunizations	+/0/+/0/+				

												Td/Tdap immunizations	0/0/0/0/0				

												HbA1c testing		0/0/0/0/0			

												Medical attention for nephropathy		0/0/0/0/+			

88	2021	Zhang	QN	USA	Commercial ACO	HMO, large independent practice association of physicians and hospital system	Global payment + shared savings	To	Enrolled members of commercial HMO	11,958 patients	20,275 patients	Generic drug use (2010/11/12/13/14)		0/0/0/0/0			

												Generic drug spending (2010/11/12/13/14)			0/+/0/0/0		

												Brand drug use (2010/11/12/13/14)		0/0/0/0/0			

												Brand drug spending (2010/11/12/13/14)			0/0/0/0/0		

												Total prescription drug use (2010/11/12/13/14)		0/0/+/+/0			

												Total prescription drug spending (2010/11/12/13/14)			0/0/0/0/0		

												Medication adherence (2010/11/12/13/14)	–/–/–/0/0				

74	2020	Marrufo	QN	USA	ESRD Seamless Care Organization (ESCO)	Dialysis facilities, nephrologists, and other providers	Global payment + shared savings	To	Medicare fee-for-service beneficiaries	73,094 beneficiaries	60,464 beneficiaries	Radiation therapy use (180 days/30 days prior to death)		0/0			B1

												Chemotherapy use (180 days/30 days prior to death)		0/0			

												Hospice use (180 days/30 days prior to death)		0/0			

												ESRD hospitalization complications payment			+		

												Total dialysis payment			–		

												Hospitalizations		+			

												Readmissions		0			

												ED visits		0			

												Emergency dialysis		0			

												Dialysis sessions		+			

												Catheter placement	+				

												Vascular access complications	0				


### Performance of Care Networks

In general, the results of the studies show that payment models have diverse effects on the performance of a network.

#### Capitation

From the studies, it can be concluded that a capitation approach, both stand-alone or in combination with elements of risk-and-gain-sharing or P4P, is an effective payment model to reduce spending [42] and improve most types of health care utilization [42,43,44,45], without affecting the quality of care [45]. With regard to utilization, both timely discharge and the length of home health episodes showed the desired increase, and inpatient hospital admissions decreased as was anticipated [42,44,45]. Most visit types were positively impacted for home health beneficiaries and community-dwelling elderly: emergency department (ED) hospital visits and home health visits decreased, whereas office-based and preventive visits increased [42,45]. However, HMO enrollees experienced an unwanted decrease in physician visits [43]. No effects were found for one prevention activity (colonoscopy screening) and hospital readmission rates [45].

#### Disease-based Bundled Payments

Four out of five of the studies that considered disease-based bundled payments to the network, had a focus on diabetes management programs [46,47,48,49]. In terms of utilization, use of specialist care decreased as expected and hoped for, but eye testing also decreased, and this had not been an intended outcome. All other measures of medical testing increased as was envisioned [47]. Furthermore, the use of institutional postdischarge facilities was successfully reduced [50]. The model negatively impacted performance on total spending, medical specialist and medication spending, but post discharge spending and primary care spending were curbed [46,47,49,50]. One qualitative study [48] mapped other consequences and found some positive effects (better collaboration, greater transparency, and better process quality) but also some negative ones (increased administrative burden, greater price variations, and unwanted dominance by GP care groups). Quality indicators were identified in one study, indicating no significant effect on mortality and a desired decrease in readmissions, with the exception that readmissions for medical episodes were not significantly affected if the bundled payment was not in the setting of an ACO [50].

#### Pay-for-performance

Of the eight studies on P4P, one described P4P as a means to reimburse on the network level [51], one focused on payment flows both within and to the network [52], while, in the rest of the studies, P4P was used to make disbursements to individual providers in the network. Levin-Scherz et al. [51] only studied the utilization of diabetes-related services: screening and testing were successfully intensified, but a form of asthma therapy was unaffected. The results from the seven other studies are mixed in terms of both quality and utilization [44,52,53,54,55,56,57]. Marton et al. [44] observed an unsought increase in the utilization of health care professionals, whereas utilization of outpatient clinics and length of stay were successfully reduced. Substance use disorder (SUD) screening, blood lead level screening and visits that focus on prevention (well care visits) increased as hoped. However, treatments for ADHD and SUD were not affected [54,56]. An overall composite measure of quality showed desired improvements [53], but a more detailed look reveals that the prevalence of asthma, pharyngitis, upper respiratory infection, and rotavirus were not affected, and the performance related to several types of immunizations varied widely [56]. Spending was investigated in one study, which found no significant effects on shared savings or outpatient spending [57].

#### Global payment with shared savings

Under this payment model, quality tended to improve and, if not, to remain stable [40,58,59,60,61,62,63,64,65,66,67,68,69,70,71,72,73,74,75]. The same was true for spending [59,60,61,66,67,68,72,73,74,75,76,77,78,79,80,81,82,83,84,85,86,87,88,89], whereas the effects on utilization were more diverse [41,58,59,60,61,63,67,68,72,74,75,76,82,83,84,86,88,90,91,92,93,94,95,96,97,98,99,100]. Although quality improved overall, some negative outcomes could be observed. For instance, the percentage of patients that met the quality indicator for LDL-cholesterol testing and the number of people identified as having a depressive disorder had not improved, the latter hinting at an under-detection of depressive disorders [66,71]. Furthermore, medication adherence deteriorated in the first three years after payment model implementation, and adequate care for patients with depression was also negatively affected [88,100].

Findings related to spending performance were clearly mixed. Some studies indicated that spending was successfully curbed overall [59,73,74,85], whereas other studies showed no improvements in general [66,71,77,79,84,86,87,88]. McWilliams et al. [68] found a more nuanced situation: declining spending rates for networks adopting this payment model in 2012 but not in those starting in 2013. These effects of the timing when a network adopts the model are visible specifically in the spending trends of hospital-integrated ACOs (as opposed to physician group ACOs) and for skilled nursing facilities [80,81]. Overall, shared savings arrangements with increased risk exposure show a more positive effect on spending than arrangements with less provider risk [67,72,82]. For arrangements with increased risk exposure, the differences in spending performance could be explained by the number of years using, and hence experience with, the model [66,72] and also by spending category (Medicare part D or A/B spending) [83].

Performance in terms of utilization varied widely, especially for visits and hospitalizations [58,59,60,72,96]. Some differences in visit rates seem to be explained by location and ACO-orientation (primary care or specialty-oriented) [60,96]. Furthermore, use of low-value care (i.e., care that does not or only minimally benefits patients) was not affected according to Modi et al. [94] whereas Schwartz et al. [82] did show favourable reductions. Heightened levels of provider risk did seem to play an important role in increasing testing: some studies showed that the amount of testing was successfully increased [59,67], although others contradicted this [68]. Findings on performance in terms of screening for breast cancer are contradictory. One study [93] observed an unwanted decrease in mammography screening, whereas other studies demonstrate desirable increases in screening [75] or appropriate screening (which refers to the practice of increasing screening rates for patients likely to benefit and decreasing screening rates for those unlikely to benefit) [41,95]. Rates for other types of cancer screening (cervical, prostate and colorectal) were successfully increased [75,93,95].

For all three categories (quality, utilization, and spending), indicator-level differences are in part attributable to geographical state [60], entry cohort [66,68,80,81,89,96,97], and performance year [66,72,75,88,89]. It was observed that performance does not necessarily improve with time, the effects may slip back from one year to the next. In terms of utilization, the type of disease that is being screened for [93,95] or the type of low-value service [82] seem to explain indicator-specific differences. Differences in quality at the indicator level (e.g., the number of readmissions) can be linked to the type of surgical procedure [61] or to the level of risk [66,69]. In shared savings arrangements with little provider risk, two of the ten measures of patient experience improved whereas, when there were higher levels of risk, improvements in patient experience were lacking [101]. Concerning other consequences, the proportion of vulnerable patients served by physician groups was not significantly changed, neither was the adoption of novel technologies for six surgical procedures [99,102].

#### Global payment with shared savings and pay-for-performance

This payment model led to some improvement in utilization rates [103,104,105], in quality [103,106,107,108,109,110,111], and in spending [105,106,107,108,109]. Utilization did improve for tobacco cessation treatment with increased use of related therapies and drug regimens [104]. In contrast, with the exception of LDL-cholesterol testing, this model had no effect on testing and screening, overall drug utilization, and admission rates for ambulatory-care-sensitive conditions (ACSCs) [103,106,112,113]. The model’s effects on substance use disorder services depended on the patient population [103]. The majority of quality indicators showed positive results. Adult preventive care quality (an aggregate indicator for several screening measures and antibiotic use) improved over time [107,108,109] and Chien et al. [110] revealed that quality in terms of measures linked to P4P improved but that no effects were observed for quality measures not tied to P4P. Except for patients up until 21 years of age, total medical spending was successfully contained under this payment model [110]. For specific spending indicators, the findings varied, with SUD spending and drug spending trends unaffected [103,112]. Turning to other consequences, Blewett et al. [114] showed that adopting this payment model in the setting of the Integrated Health Partnership in Minnesota led to the forming of community partnerships and service integration.

#### Global payment with shared savings and pay-for-coordination

Only one study, on the Total Cost and Care Improvement (TCCI) initiative, investigates a model that combined a global payment with shared savings and pay-for-coordination. Afendulis et al. [115] showed that this specific model had no effects on either utilization or spending, while quality was not investigated.

## Discussion

This review compiles the current evidence on the effect of various network-level payment models on the performance of care networks. The empirical results on performance for a set of payment models are mixed. Overall, no single payment model was associated with consistent improvements in network performance on all three criteria categories (utilization, spending, and quality). However, a more detailed look at the individual categories reveals some insights. First concerning quality, the papers reviewed found that, depending on the quality indicator investigated, quality generally increased or at least remained stable under whichever payment model they were investigating. The same can be said for utilization. Furthermore, all but two payment models showed improved performance in terms of spending. A negative effect on spending performance was found when adopting the disease-based bundled payment model, which failed to curb spending in most instances. Looking at other consequences of these payment models for care networks, some had identified improvements in performance indicators related to collaboration. However, these conclusions were almost entirely related to the effect of making payments to the network, and the very few studies that investigated payments within the network only addressed the P4P model.

Our findings support most, but not all, of the theory-based expectations of the effects of payment models on network performance. The expectation is that, under risk-based payment models such as capitation, disease-based bundled payment, and global payment, providers will be incentivized to minimize costs, control their volume by proactively monitoring utilization and spending, and invest in prevention to curb downstream health care use [13,20,116]. However, our analysis indicates that only capitation proved able to improve performance in terms of both spending and utilization. When applying disease-based bundled payments, performance in terms of utilization improved as predicted, but spending was not contained. In their study, Mohnen et al. [46] suggest that these results could be due to the negotiated contract working out well for the provider (a high bundle price) and that the short length of their study following the introduction of the scheme might not reveal longer term effects. Turning to the global payment approach, performance in terms of spending and utilization in the various studies was found to generally improve or at least remain stable. In the studies where shared savings had been added to the basic global payment approach, we found that shared savings arrangements where there was a significant risk element showed somewhat better performance in terms of spending compared with arrangements with less risk. This finding corresponds with the view that risk sharing arrangements induce cost-conscious behaviour [117]. The payment models discussed above are, by their very nature, more focused on cost containment then on quality improvement [13,118]. This focus has the associated risk of stinting on care [12]. However, our results do not reveal any adverse effects on the quality of care: quality improved or remained stable, with no clear differences between the models.

P4P has gained much attention in the scholarly literature as it is expected to enhance performance by financially incentivizing providers to deliver the best care. However, the evidence from our analysis is not consistently positive, a finding that is in line with earlier reviews of P4P [119,120]. Further, our results do not convincingly demonstrate that P4P has added value over approaches based on a global payment plus shared savings. That is, no meaningful performance differences could be discerned between global payment plus shared savings arrangements with or without additional P4P. Cattel and Eijkenaar [8] offered a potential explanation for this: that P4P is only a small part of the total reimbursement received by a provider. Following this line of reasoning, the P4P incentive in relation to global payment plus shared savings might thus have been too small to have a significant impact on performance.

Also, our results show that the relation between payment models and effects is not necessarily stable but depends on several other factors. For instance, our results suggest that the cohort entry year (starting year of the payment model), scope of services explain differences in performance, and timing of the performance assessment (years since implementation of a payment model). In terms of entry cohort, our review shows that early ACO entrants seem to do better overall in improving performance. Related to this, McWilliams et al. [81] found that, for ACOs offering a wide range of services (hospital-integrated ACOs) – but not for narrow-scoped ACOs – there were performance differences between early and late adopters. Others have also identified scope of services as one of eight organizational attributes that might possibly explain performance differences between early and late adopters, alongside other attributes such as prior experience with payment reform [121,122]. In terms of changes in the years following the introduction of network payments, it seems that initial performance improvements tail off in later years. Thus, improvements might not continue and may even recede as time goes by. These studies that give insight in performance on the longer term, have a maximum span of three to five years. Other than this, evidence on the sustainability of incentives that derive from the payment models is lacking. More research on incentive sustainability and, accordingly, longer term impact on performance is warranted. Next to ‘how long’ performance is observed, it is important to emphasize ‘what’ performance is observed, or, neglected. Except for indicators of quality, patient-reported experience and outcome measures (PREMs and PROMs) have hardly been encountered in our study. As such, it can be argued whether the patient perspective is sufficiently covered in the indicators.

This review has several limitations. First, the insights are mainly drawn from studies in the USA. ACOs were formed after the passing of the Affordable Care Act in 2010 as an instrument to improve patient care but also to reduce costs, in order to tackle the ‘affordability crisis’ of the US health system [123]. This context might possibly explain the focus of the USA setting in our review, which limits generalizability. Another limitation is that the implementation of alternative payment models was generally part of a myriad of concurrent interventions, making it difficult to disentangle the effect of a payment model from those associated with other interventions. Additionally, the studies that investigated non-commercial ACOs (Medicare Shared Savings Program and Pioneer) were not explicit as to whether the risks associated with shared savings were one- or two-sided. Hence, we cannot draw any inferences on the relation between the sidedness of risk and performance.

It seemed that networks are generally able to improve their performance under the investigated payment models, it only occasionally remained unchanged and rarely deteriorated. It would be valuable to investigate what circumstances are required to achieve a certain performance. This aspect was emphasized by Kaufman et al. [15, p.270] who state that “looking at outcomes alone misses important information regarding what it takes to produce those outcomes”. Here, further research could adopt a mixed-methods approach, combining qualitative research, to uncover contexts, mechanisms, and interpersonal dynamics within networks, with quantitative methods that measure quality, utilization, and spending outcomes on the network level. This contextual and interpersonal perspective would be a valuable addition to studies that have comprehensively investigated the more technical aspects of payment reform such as key design features of payment models [14,124,125]. Furthermore, although bundled payment evaluations are omnipresent in the literature, more research is needed into multi-provider bundled payments, as most evaluations focus on single provider bundled payments. Additionally, to date, provider participation in reformed payment methods is largely voluntary, although policymakers are exploring the possibilities of mandatory participation [126]. Developing a ‘theory-based understanding’ [127] of contexts and mechanisms – payment being one of many mechanisms [128] – under which certain outcomes are produced could help providers prepare for future, possibly mandatory, payment reform.

## Conclusion

The aim of this study was to unravel the effects that network-level payment models have on the multidimensional (quality, utilization, spending, *other)* performance concept in care networks. Although network-level reimbursement schemes are still in their infancy, our review shows that network-level payment has the potential to improve network performance. Given that health care networks are becoming increasingly common, it seems fruitful to continue experimenting with network-level payment models. In future studies, it will be important to broaden the scope beyond only outcomes and to also take contexts and the mechanisms through which networks adopt and implement payment models into account.

## Additional File

The additional file for this article can be found as follows:

10.5334/ijic.6002.s1 Supplementary File. Search strings.

## Footnotes

For this study, first symbol indicates results of comparison with control group that received exclusively fee-for-service payments, second symbol indicates results of comparison with control group that were salaried.

For this study, first symbol indicates results for patients facing behavioral health risks and second symbol indicates results for patients not facing behavioral health risks.

This study has also stratified post-hoc for ethnicity/disparity.

## References

[1] Amelung V, Stein V, Goodwin N, Balicer R, Nolte E, Suter E. Handbook Integrated Care. Amelung V, Stein V, Goodwin N, Balicer R, Nolte E, Suter E (eds.), Handbook Integrated Care. Cham: Springer International Publishing 2017; 1–595 DOI: 10.1007/978-3-319-56103-5

[2] Raus K, Mortier E, Eeckloo K. Organizing Health Care Networks: Balancing Markets, Government and Civil Society. International Journal of Integrated Care. 2018 Jul 11; 18(3): 1–7. DOI: 10.5334/ijic.3960 PMC6078116 30093844

[3] Sheaff R, Benson L, Farbus L, Schofield J, Mannion R, Reeves D. Network resilience in the face of health system reform. Social Science & Medicine. 2010 Mar; 70(5): 779–86. DOI: 10.1016/j.socscimed.2009.11.011 20056304

[4] Miller RH. Health System Integration: A Means to an End. Health Affairs. 1996 Jan; 15(2): 92–106. DOI: 10.1377/hlthaff.15.2.92 8690393

[5] Sheaff R, Schofield J. Inter-Organizational Networks in Health Care. Ferlie E, Montgomery K, Reff Pedersen A (eds.), Vol. 1. Oxford University Press. 2016; 1–27. DOI: 10.1093/oxfordhb/9780198705109.013.29

[6] WHO. The world health report: health systems financing: the path to universal coverage; 2010. 10.2471/BLT.10.078741 PMC2878164 20539847

[7] Miller HD. From Volume To Value: Better Ways To Pay For Health Care. Health Affairs. 2009 Sep; 28(5): 1418–28. DOI: 10.1377/hlthaff.28.5.1418 19738259

[8] Cattel D, Eijkenaar F. Value-Based Provider Payment Initiatives Combining Global Payments With Explicit Quality Incentives: A Systematic Review. Medical Care Research and Review. 2019 Jun 19;107755871985677. DOI: 10.1177/1077558719856775 PMC7536531 31216945

[9] Ginsburg PB, Grossman JM. When the price isn’t right: how inadvertent payment incentives drive medical care. Health Affairs. 2005; Suppl Web(August): 376–84. DOI: 10.1377/hlthaff.W5.376 16091408

[10] Stokes J, Struckmann V, Kristensen SR, Fuchs S, van Ginneken E, Tsiachristas A, et al. Towards incentivising integration: A typology of payments for integrated care. Health Policy. 2018 Sep; 122(9): 963–9. DOI: 10.1016/j.healthpol.2018.07.003 30033204

[11] Conrad DA, Perry L. Quality-Based Financial Incentives in Health Care: Can We Improve Quality by Paying for It? Annual Review of Public Health. 2009 Apr; 30(1): 357–71. DOI: 10.1146/annurev.publhealth.031308.100243 19296779

[12] Hubley SH, Miller BF. Implications of Healthcare Payment Reform for Clinical Psychologists in Medical Settings. Journal of Clinical Psychology in Medical Settings. 2016 Mar 26; 23(1): 3–10. DOI: 10.1007/s10880-016-9451-1 26916051

[13] Barnum H, Kutzin J, Saxenian H. Incentives and provider payment methods. International Journal of Health Planning and Management. 1995 Jan; 10(1): 23–45. DOI: 10.1016/B978-012373960-5.00173-8 10142120

[14] Vlaanderen FP, Tanke MA, Bloem BR, Faber MJ, Eijkenaar F, Schut FT, et al. Design and effects of outcome-based payment models in healthcare: a systematic review. The European Journal of Health Economics. 2019 Mar 5; 20(2): 217–32. DOI: 10.1007/s10198-018-0989-8 29974285 PMC6438941

[15] Kaufman BG, Spivack BS, Stearns SC, Song PH, O’Brien EC. Impact of Accountable Care Organizations on Utilization, Care, and Outcomes: A Systematic Review. Medical Care Research and Review. 2019 Jun 12; 76(3): 255–90. DOI: 10.1177/1077558717745916 29231131

[16] Provan KG, Kenis P. Modes of Network Governance: Structure, Management, and Effectiveness. Journal of Public Administration Research and Theory. 2007 Jun 29; 18(2): 229–52. DOI: 10.1093/jopart/mum015

[17] Tsiachristas A. Payment models for integrated care. 18th International Conference on Integrated Care. Utrecht, The Netherlands; 2018.

[18] Quinn K. The 8 Basic Payment Methods in Health Care. Annals of Internal Medicine. 2015 Aug 18; 163(4): 300. DOI: 10.7326/M14-2784 26259075

[19] Quinn K. Achieving cost control, care coordination, and quality improvement in the medicaid program. Journal of Ambulatory Care Management. 2010; 33(1): 38–49. DOI: 10.1097/jac.0b013e3181cfc12a 20026997

[20] Frakt AB, Mayes R. Beyond Capitation: How New Payment Experiments Seek To Find The ‘Sweet Spot’ In Amount Of Risk Providers And Payers Bear. Health Affairs. 2012 Sep; 31(9): 1951–8. DOI: 10.1377/hlthaff.2012.0344 22949443

[21] Conrad DA. The Theory of Value-Based Payment Incentives and Their Application to Health Care. Health Services Research. 2015 Dec; 50: 2057–89. DOI: 10.1111/1475-6773.12408 26549041 PMC5338202

[22] Burns LR, Pauly MV. Transformation of the Health Care Industry: Curb Your Enthusiasm? The Milbank Quarterly. 2018 Mar; 96(1): 57–109. DOI: 10.1111/1468-0009.12312 29504199 PMC5835686

[23] Kahneman D, Tversky A. Prospect Theory: An Analysis of Decision under Risk. Econometrica. 1979 Mar; 47(2): 263. DOI: 10.2307/1914185

[24] Nembhard IM, Tucker AL. Applying Organizational Learning Research to Accountable Care Organizations. Medical Care Research and Review. 2016; 73(6): 673–84. DOI: 10.1177/1077558716640415 27034437

[25] Newhouse JP. Risk Adjustment, Market Equilibrium, and Carveouts. In: Pricing the Priceless. The MIT Press; 2002. Available from: https://direct.mit.edu/books/book/2692/chapter/72797/risk-adjustment-market-equilibrium-and-carveouts.

[26] Eijkenaar F. Key issues in the design of pay for performance programs. European Journal of Health Economics. 2013; 14(1): 117–31. DOI: 10.1007/s10198-011-0347-6 PMC3535413 21882009

[27] Gaynor M, Rebitzer JB, Taylor LJ. Physician Incentives in Health Maintenance Organizations. Journal of Political Economy. 2004 Aug; 112(4): 915–31. DOI: 10.1086/421172

[28] Valentijn PP, Schepman SM, Opheij W, Bruijnzeels MA. Understanding integrated care: a comprehensive conceptual framework based on the integrative functions of primary care. International Journal of Integrated Care. 2013 Mar 22; 13(1). DOI: 10.5334/ijic.886 PMC3653278 23687482

[29] Rischatsch M. Who joins the network? Physicians’ resistance to take budgetary co-responsibility. Journal of Health Economics. 2015 Mar; 40: 109–21. DOI: 10.1016/j.jhealeco.2014.12.002 25637711

[30] Levac D, Colquhoun H, O’Brien KK. Scoping studies: advancing the methodology. Implementation Science. 2010 Dec 20; 5(1): 69. DOI: 10.1186/1748-5908-5-69 20854677 PMC2954944

[31] Peters MDJ, Godfrey CM, Khalil H, McInerney P, Parker D, Soares CB. Guidance for conducting systematic scoping reviews. International Journal of Evidence-Based Healthcare. 2015 Sep; 13(3): 141–6. DOI: 10.1097/xeb.0000000000000050 26134548

[32] Tricco AC, Lillie E, Zarin W, O’Brien KK, Colquhoun H, Levac D, et al. PRISMA Extension for Scoping Reviews (PRISMA-ScR): Checklist and Explanation. Annals of Internal Medicine. 2018 Oct 2; 169(7): 467. DOI: 10.7326/M18-0850 30178033

[33] Arksey H, O’Malley L. Scoping studies: towards a methodological framework. International Journal of Social Research Methodology. 2005 Feb; 8(1): 19–32. DOI: 10.1080/1364557032000119616

[34] Minkman M, Ahaus K, Huijsman R. Performance improvement based on integrated quality management models: what evidence do we have? A systematic literature review. International Journal for Quality in Health Care. 2007 Apr 1; 19(2): 90–104. DOI: 10.1093/intqhc/mzl071 17277010

[35] Bramer WM. Serving Evidence Syntheses: Improving literature retrieval in systematic reviews. Erasmus University Rotterdam; 2019. Available from: https://repub.eur.nl/pub/120107.

[36] OECD. Public Health Reviews [Internet]. [cited 2021 December 23] Available from: https://www.oecd.org/health/public-health-reviews.htm.

[37] Moher D, Liberati A, Tetzlaff J, Altman DG. Preferred Reporting Items for Systematic Reviews and Meta-Analyses: The PRISMA Statement. PLoS Medicine. 2009 Jul 21; 6(7): e1000097. DOI: 10.1371/journal.pmed.1000097 19621072 PMC2707599

[38] Clarivate Analytics. EndNote X9 Quick Reference Guide for Mac. 2018.

[39] Agarwal R, Liao JM, Gupta A, Navathe AS. The Impact Of Bundled Payment On Health Care Spending, Utilization, And Quality: A Systematic Review. Health Affairs. 2020 Jan 1; 39(1): 50–7. DOI: 10.1377/hlthaff.2019.00784 31905061

[40] Fraze TK, Lewis VA, Tierney E, Colla CH. Quality of Care Improves for Patients with Diabetes in Medicare Shared Savings Accountable Care Organizations: Organizational Characteristics Associated with Performance. Population Health Management. 2018 Oct; 21(5): 401–8. DOI: 10.1089/pop.2017.0102 29211623 PMC6425920

[41] Narayan AK, Harvey SC, Durand DJ. Impact of medicare shared savings program accountable care organizations at screening mammography: A retrospective cohort study. Radiology. 2017; 282(2): 437–42. DOI: 10.1148/radiol.2016160554 27646860

[42] Schlenker RE, Shaughnessy PW, Hittle DF. Patient-level cost of home health care under capitated and fee-for-service payment. Inquiry. 1995; 32(3): 252–70. 7591040

[43] Robinson JC, Casalino LP. The Growth of Medical Groups Paid through Capitation in California. New England Journal of Medicine. 1995; 333(25): 1684–7. DOI: 10.1056/NEJM199512213332506 7477222

[44] Marton J, Yelowitz A, Talbert JC. A tale of two cities? The heterogeneous impact of medicaid managed care. Journal of Health Economics. 2014 Jul; 36(1): 47–68. DOI: 10.1016/j.jhealeco.2014.03.001 24747920

[45] Mandal AK, Tagomori GK, Felix R V., Howell SC. Value-based contracting innovated Medicare advantage healthcare delivery and improved survival. American Journal of Managed Care. 2017 Feb 1; 23(2): e41–9. 28245661

[46] Mohnen S, Baan C, Struijs J. Bundled Payments for Diabetes Care and Healthcare Costs Growth: A 2-Year Follow-up Study. American Journal of Accountable Care. 2015; 63–70.

[47] Busse R, Stahl J. Integrated Care Experiences And Outcomes In Germany, The Netherlands, And England. Health Affairs. 2014 Sep; 33(9): 1549–58. DOI: 10.1377/hlthaff.2014.0419 25201659

[48] de Bakker DH, Struijs JN, Baan CA, Raams J, de Wildt J-E, Vrijhoef HJM, et al. Early Results From Adoption Of Bundled Payment For Diabetes Care In The Netherlands Show Improvement In Care Coordination. Health Affairs. 2012 Feb; 31(2): 426–33. DOI: 10.1377/hlthaff.2011.0912 22323174

[49] Karimi M, Tsiachristas A, Looman W, Stokes J, Galen M van, Rutten-van Mölken M. Bundled payments for chronic diseases increased health care expenditure in the Netherlands, especially for multimorbid patients. Health Policy. 2021; 125(6): 751–9. DOI: 10.1016/j.healthpol.2021.04.004 33947604

[50] Navathe AS, Liao JM, Wang E, Isidro U, Zhu J, Cousins DS, et al. Association of Patient Outcomes With Bundled Payments Among Hospitalized Patients Attributed to Accountable Care Organizations. JAMA Health Forum. 2021; 2(8): e212131. DOI: 10.1001/jamahealthforum.2021.2131 PMC8796940 35977188

[51] Levin-Scherz J, DeVita N, Timbie J. Impact of pay-for-performance contracts and network registry on diabetes and asthma HEDIS® measures in an integrated delivery network. Medical Care Research and Review. 2006; 63(1 SUPPL.): 14–28. DOI: 10.1177/1077558705284057 16688922

[52] Mandel KE, Kotagal UR. Pay for performance alone cannot drive quality. Archives of Pediatrics and Adolescent Medicine. 2007; 161(7): 650–5. DOI: 10.1001/archpedi.161.7.650 17606827

[53] Atkinson GJ, Masiulis KE, Felgner L, Schumacher DN. Provider-Initiated Pay-for-Performance in a Clinically Integrated Hospital Network. Journal For Healthcare Quality. 2010 Jan; 32(1): 42–50. DOI: 10.1111/j.1945-1474.2009.00063.x 20151591

[54] Rieckmann T, Renfro S, McCarty D, Baker R, McConnell KJ. Quality Metrics and Systems Transformation: Are We Advancing Alcohol and Drug Screening in Primary Care? Health Services Research. 2018 Jun; 53(3): 1702–26. DOI: 10.1111/1475-6773.12716 28568245 PMC5980159

[55] Hibbard JH, Greene J, Sacks R, Overton V. Does compensating primary care providers to produce higher quality make them more or less patient centric? Medical Care Research and Review. 2015; 72(4): 481–95. DOI: 10.1177/1077558715586291 25962744

[56] Gleeson S, Kelleher K, Gardner W. Evaluating a Pay-for-Performance Program for Medicaid Children in an Accountable Care Organization. JAMA Pediatrics. 2016 Mar 1; 170(3): 259. DOI: 10.1001/jamapediatrics.2015.3809 26810378

[57] Ganguli I, Lupo C, Mainor AJ, Orav EJ, Blanchfield BB, Lewis VA, et al. Association between specialist compensation and Accountable Care Organization performance. Health Services Research. 2020 Oct 27; 55(5): 722–8. DOI: 10.1111/1475-6773.13323 32715464 PMC7518824

[58] Sandberg SF, Erikson C, Owen R, Vickery KD, Shimotsu ST, Linzer M, et al. Hennepin health: A safety-net accountable care organization for the expanded medicaid population. Health Affairs. 2014; 33(11): 1975–84. DOI: 10.1377/hlthaff.2014.0648 25367993

[59] Pope G, Kautter J, Leung M, Trisolini M, Adamache W, Smith K. Financial and quality impacts of the medicare Physician Group practice demonstration. Medicare and Medicaid Research Review. 2014; 4(3). DOI: 10.5600/mmrr.004.03.a01 PMC4144360 25161812

[60] Rutledge RI, Romaire MA, Hersey CL, Parish WJ, Kissam SM, Lloyd JT. Medicaid Accountable Care Organizations in Four States: Implementation and Early Impacts. Milbank Quarterly. 2019; 97(2): 583–619. DOI: 10.1111/1468-0009.12386 PMC6554509 30957294

[61] Borza T, Oerline MK, Skolarus TA, Norton EC, Dimick JB, Jacobs BL, et al. Association between Hospital Participation in Medicare Shared Savings Program Accountable Care Organizations and Readmission Following Major Surgery. Annals of Surgery. 2019; 269(5): 873–8. DOI: 10.1097/SLA.0000000000002737 29557880 PMC6146076

[62] Winblad U, Mor V, McHugh JP, Rahman M. ACO-Affiliated Hospitals Reduced Rehospitalizations From Skilled Nursing Facilities Faster Than Other Hospitals. Health Affairs. 2017 Jan; 36(1): 67–73. DOI: 10.1377/hlthaff.2016.0759 28069848 PMC5553196

[63] Kaufman BG, O’Brien EC, Stearns SC, Matsouaka R, Holmes GM, Weinberger M, et al. The Medicare Shared Savings Program and Outcomes for Ischemic Stroke Patients: a Retrospective Cohort Study. Journal of General Internal Medicine. 2019; 34(12): 2740–8. DOI: 10.1007/s11606-019-05283-1 31452032 PMC6854149

[64] Bain AM, Werner RM, Yuan Y, Navathe AS. Do hospitals participating in accountable care organizations discharge patients to higher quality nursing homes? Journal of Hospital Medicine. 2019; 14(5): 288–9. DOI: 10.12788/jhm.3147 30897056 PMC7172035

[65] Kim Y, Thirukumaran CP, Li Y. Greater reductions in readmission rates achieved by urban hospitals participating in the medicare shared savings program. Medical Care. 2018; 56(8): 686–92. DOI: 10.1097/mlr.0000000000000945 29912839

[66] Busch AB, Huskamp HA, McWilliams JM. Early efforts by medicare accountable care organizations have limited effect on mental illness care and management. Health Affairs. 2016; 35(7): 1247–56. DOI: 10.1377/hlthaff.2015.1669 27385241

[67] McWilliams JM, Chernew ME, Landon BE, Schwartz AL. Performance Differences in Year 1 of Pioneer Accountable Care Organizations. New England Journal of Medicine. 2015; 372(20): 1927–36. DOI: 10.1056/NEJMsa1414929 PMC4475634 25875195

[68] McWilliams JM, Hatfield LA, Chernew ME, Landon BE, Schwartz AL. Early Performance of Accountable Care Organizations in Medicare. New England Journal of Medicine. 2016; 374(24): 2357–66. DOI: 10.1056/NEJMsa1600142 PMC4963149 27075832

[69] Duggal R, Zhang Y, Diana ML. The Association Between Hospital ACO Participation and Readmission Rates. Journal of Healthcare Management. 2018 Sep; 63(5): e100–14. DOI: 10.1097/JHM-D-16-00045 30180036

[70] Herrel LA, Norton EC, Hawken SR, Ye Z, Hollenbeck BK, Miller DC. Early impact of Medicare accountable care organizations on cancer surgery outcomes. Cancer. 2016 Sep 1; 122(17): 2739–46. DOI: 10.1002/cncr.30111 27218198 PMC4992435

[71] Markovitz AA, Hollingsworth JM, Ayanian JZ, Norton EC, Yan PL, Ryan AM. Performance in the Medicare Shared Savings Program After Accounting for Nonrandom Exit. Annals of Internal Medicine. 2019 Jul 2; 171(1): 27. DOI: 10.7326/M18-2539 31207609 PMC8757576

[72] Nyweide DJ, Lee W, Cuerdon TT, Pham HH, Cox M, Rajkumar R, et al. Association of Pioneer Accountable Care Organizations vs traditional Medicare fee for service with spending, utilization, and patient experience. JAMA. 2015; 313(21): 2152–61. DOI: 10.1001/jama.2015.4930 25938875

[73] Colla CH, Lewis VA, J. Gottlieb D, Fisher ES. Cancer spending and accountable care organizations: Evidence from the physician group practice demonstration. Healthcare. 2013; 1(3–4): 100–7. DOI: 10.1016/j.hjdsi.2013.05.005 PMC4110916 25072017

[74] Marrufo G, Colligan EM, Negrusa B, Ullman D, Messana J, Shah A, et al. Association of the Comprehensive End-Stage Renal Disease Care Model With Medicare Payments and Quality of Care for Beneficiaries With End-Stage Renal Disease. JAMA Internal Medicine. 2020 Jun 1; 180(6): 852. DOI: 10.1001/jamainternmed.2020.0562 32227133 PMC7105949

[75] Zhang H, Cowling DW, Graham JM, Taylor E. Five-year Impact of a Commercial Accountable Care Organization on Health Care Spending, Utilization, and Quality of Care. Medical Care. 2019 Nov; 57(11): 845–54. DOI: 10.1097/MLR.0000000000001179 31348124

[76] Borza T, Kaufman SR, Yan P, Herrel LA, Luckenbaugh AN, Miller DC, et al. Early effect of Medicare Shared Savings Program accountable care organization participation on prostate cancer care. Cancer. 2018 Feb 1; 124(3): 563–70. DOI: 10.1002/cncr.31081 29053177 PMC5781227

[77] Cole AP, Krasnova A, Ramaswamy A, Friedlander DF, Fletcher SA, Sun M, et al. Prostate cancer in the medicare shared savings program: are Accountable Care Organizations associated with reduced expenditures for men with prostate cancer? Prostate Cancer and Prostatic Diseases. 2019; 22(4): 593–9. DOI: 10.1038/s41391-019-0138-1 30980025

[78] Colla CH, Lewis VA, Kao L-S, O’Malley AJ, Chang C-H, Fisher ES. Association Between Medicare Accountable Care Organization Implementation and Spending Among Clinically Vulnerable Beneficiaries. JAMA Internal Medicine. 2016 Aug 1; 176(8): 1167. DOI: 10.1001/jamainternmed.2016.2827 27322485 PMC4969198

[79] Lam MB, Figueroa JF, Zheng J, Orav EJ, Jha AK. Spending among patients with cancer in the first 2 years of accountable care organization participation. Journal of Clinical Oncology. 2018; 36(29): 2955–60. DOI: 10.1200/jco.18.00270 30156985

[80] McWilliams JM, Gilstrap LG, Stevenson DG, Chernew ME, Huskamp HA, Grabowski DC. Changes in postacute care in the medicare shared savings program. JAMA Internal Medicine. 2017; 177(4): 518–26. DOI: 10.1001/jamainternmed.2016.9115 28192556 PMC5415671

[81] McWilliams JM, Hatfield LA, Landon BE, Hamed P, Chernew ME. Medicare Spending after 3 Years of the Medicare Shared Savings Program. New England Journal of Medicine. 2018; 379(12): 1139–49. DOI: 10.1056/nejmsa1803388 PMC6269647 30183495

[82] Schwartz AL, Chernew ME, Landon BE, Michael McWilliams J. Changes in low-value services in year 1 of the medicare pioneer accountable care organization program. JAMA Internal Medicine. 2015; 175(11): 1815–25. DOI: 10.1001/jamainternmed.2015.4525 26390323 PMC4928485

[83] Zhang Y, Caines KJ, Powers CA. Evaluating the effects of pioneer accountable care organizations on medicare part D drug spending and utilization. Medical Care. 2017; 55(5): 470–5. DOI: 10.1097/mlr.0000000000000686 28060052

[84] Lam MB, Zheng J, Orav EJ, Jha AK. Early Accountable Care Organization Results in End-of-Life Spending Among Cancer Patients. JNCI: Journal of the National Cancer Institute. 2019 Dec 1; 111(12): 1307–13. DOI: 10.1093/jnci/djz033 30859226 PMC6910163

[85] Bakre S, Hollingsworth JM, Yan PL, Lawton EJ, Hirth RA, Shahinian VB. Accountable Care Organizations and Spending for Patients Undergoing Long-Term Dialysis. Clinical Journal of the American Society of Nephrology. 2020 Dec 7; 15(12): 1777–84. DOI: 10.2215/CJN.02150220 33234541 PMC7769034

[86] Chang C-H, Mainor A, Colla C, Bynum J. Utilization by Long-Term Nursing Home Residents Under Accountable Care Organizations. Journal of the American Medical Directors Association. 2021 Feb; 22(2): 406–12. DOI: 10.1016/j.jamda.2020.05.055 32693998

[87] Erfani P, Phelan J, Orav EJ, Figueroa JF, Jha AK, Lam MB. Spending outcomes among patients with cancer in accountable care organizations 4 years after implementation. Cancer. 2021 Nov 12; 1–8. DOI: 10.1002/cncr.34022 PMC8837672 34767638

[88] Zhang H, Cowling DW, Graham JM, Taylor E. Impact of a commercial accountable care organization on prescription drugs. Health Services Research. 2021 Aug 28; 56(4): 592–603. DOI: 10.1111/1475-6773.13626 33508877 PMC8313955

[89] McWilliams JM, Hatfield LA, Landon BE, Chernew ME. Savings or Selection? Initial Spending Reductions in the Medicare Shared Savings Program and Considerations for Reform. The Milbank Quarterly. 2020 Sep 22; 98(3): 847–907. DOI: 10.1111/1468-0009.12468 32697004 PMC7482384

[90] Kim H, Keating NL, Perloff JN, Hodgkin D, Liu X, Bishop CE. Aggressive Care near the End of Life for Cancer Patients in Medicare Accountable Care Organizations. Journal of the American Geriatrics Society. 2019; 67(5): 961–8. DOI: 10.1111/jgs.15914 30969439 PMC13180505

[91] Colla CH, Goodney PP, Lewis VA, Nallamothu BK, Gottlieb DJ, Meara E. Implementation of a Pilot Accountable Care Organization Payment Model and the Use of Discretionary and Nondiscretionary Cardiovascular Care. Circulation. 2014 Nov 25; 130(22): 1954–61. DOI: 10.1161/CIRCULATIONAHA.114.011470 25421044 PMC4244758

[92] Trinh QD, Sun M, Krasnova A, Ramaswamy A, Cole AP, Fletcher SA, et al. Impact of Accountable Care Organizations on Prostate Cancer Screening and Biopsies in the United States. Urology Practice. 2019; 6(3): 159–64. DOI: 10.1016/j.urpr.2018.07.003 37300100

[93] Resnick MJ, Graves AJ, Gambrel RJ, Thapa S, Buntin MB, Penson DF. The association between Medicare accountable care organization enrollment and breast, colorectal, and prostate cancer screening. Cancer. 2018; 124(22): 4366–73. DOI: 10.1002/cncr.31700 30412287

[94] Modi PK, Kaufman SR, Borza T, Oliphant BW, Ryan AM, Miller DC, et al. Medicare Accountable Care Organizations and Use of Potentially Low-Value Procedures. Surgical Innovation. 2019; 26(2): 227–33. DOI: 10.1177/1553350618816594 30497340 PMC6503656

[95] Resnick MJ, Graves AJ, Thapa S, Gambrel R, Tyson MD, Lee D, et al. Medicare accountable care organization enrollment and appropriateness of cancer screening. JAMA Internal Medicine. 2018; 178(5): 648–54. DOI: 10.1001/jamainternmed.2017.8087 29554179 PMC5876897

[96] Barnett ML, McWilliams JM. Changes in specialty care use and leakage in medicare accountable care organizations. American Journal of Managed Care. 2018; 24(5): e141–9. PMC5986093 29851445

[97] McWilliams JM, Najafzadeh M, Shrank WH, Polinski JM. Association of changes in medication use and adherence with accountable care organization exposure in patients with cardiovascular disease or diabetes. JAMA Cardiology. 2017; 2(9): 1019–23. DOI: 10.1001/jamacardio.2017.2172 28700790 PMC5710170

[98] Lin Y-L, Ortiz J, Boor C. ACOsʼ Impact on Hospitalization Rates of Rural Older Adults With Diabetes. Family & Community Health. 2018; 41(4): 265–73. DOI: 10.1097/FCH.0000000000000204 30134341 PMC6107306

[99] Modi PK, Kaufman SR, Caram ME, Ryan AM, Shahinian VB, Hollenbeck BK. Medicare Accountable Care Organizations and the Adoption of New Surgical Technology. Journal of the American College of Surgeons. 2021 Feb; 232(2): 138–145.e2. DOI: 10.1016/j.jamcollsurg.2020.10.016 33122038 PMC8030123

[100] Acevedo A, Mullin BO, Progovac AM, Caputi TL, McWilliams JM, Cook BL. Impact of the Medicare Shared Savings Program on utilization of mental health and substance use services by eligibility and race/ethnicity. Health Services Research. 2021 Aug 5; 56(4): 581–91. DOI: 10.1111/1475-6773.13625 33543782 PMC8313953

[101] Diana ML, Zhang Y, Yeager VA, Stoecker C, Counts CR. The impact of accountable care organization participation on hospital patient experience. Health Care Management Review. 2019; 44(2): 148–58. DOI: 10.1097/hmr.0000000000000219 30080713

[102] Lee JT, Polsky D, Fitzsimmons R, Werner RM. Proportion of Racial Minority Patients and Patients With Low Socioeconomic Status Cared for by Physician Groups After Joining Accountable Care Organizations. JAMA Network Open. 2020 May 8; 3(5): e204439. DOI: 10.1001/jamanetworkopen.2020.4439 32383749 PMC7210481

[103] Stuart EA, Barry CL, Donohue JM, Greenfield SF, Duckworth K, Song Z, et al. Effects of accountable care and payment reform on substance use disorder treatment: evidence from the initial 3 years of the alternative quality contract. Addiction. 2017 Jan; 112(1): 124–33. DOI: 10.1111/add.13555 27517740 PMC5148657

[104] Huskamp HA, Greenfield SF, Stuart EA, Donohue JM, Duckworth K, Kouri EM, et al. Effects of Global Payment and Accountable Care on Tobacco Cessation Service Use: An Observational Study. Journal of General Internal Medicine. 2016 Oct 13; 31(10): 1134–40. DOI: 10.1007/s11606-016-3718-y 27177915 PMC5023596

[105] Hildebrandt H, Schulte T, Stunder B. Triple aim in Kinzigtal, Germany: Improving population health, integrating health care and reducing costs of care - Lessons for the UK? Journal of Integrated Care. 2012; 20(4): 205–22. DOI: 10.1108/14769011211255249

[106] McWilliams JM, Landon BE, Chernew ME. Changes in Health Care Spending and Quality for Medicare Beneficiaries Associated With a Commercial ACO Contract. JAMA. 2013 Aug 28; 310(8): 829. DOI: 10.1001/jama.2013.276302 23982369 PMC3860102

[107] Song Z, Safran DG, Landon BE, He Y, Ellis RP, Mechanic RE, et al. Health Care Spending and Quality in Year 1 of the Alternative Quality Contract. New England Journal of Medicine. 2011 Sep 8; 365(10): 909–18. DOI: 10.1056/NEJMsa1101416 PMC3526936 21751900

[108] Song Z, Safran DG, Landon B, Landrum MB, He Y, Mechanic R, et al. The ‘Alternative quality contract’ in Massachusetts, based on global budgets, lowered medical spending and improved quality. Health Affairs. 2012; 31(8): 1885–94. DOI: 10.1377/hlthaff.2012.0327 22786651 PMC3548447

[109] Song Z, Rose S, Safran DG, Landon BE, Day MP, Chernew ME. Changes in Health Care Spending and Quality 4 Years into Global Payment. New England Journal of Medicine. 2014; 371(18): 1704–14. DOI: 10.1056/NEJMsa1404026 PMC4261926 25354104

[110] Chien AT, Song Z, Chernew ME, Landon BE, McNeil BJ, Safran DG, et al. Two-Year Impact of the Alternative Quality Contract on Pediatric Health Care Quality and Spending. Pediatrics. 2014 Jan 23;133(1):96–104. DOI: 10.1542/peds.2012-3440 24366988 PMC4079291

[111] Pimperl A, Schulte T, Mühlbacher A, Rosenmöller M, Busse R, Groene O, et al. Evaluating the Impact of an Accountable Care Organization on Population Health: The Quasi-Experimental Design of the German Gesundes Kinzigtal. Population Health Management. 2017; 20(3): 239–48. DOI: 10.1089/pop.2016.0036 27565005

[112] Afendulis CC, Fendrick AM, Song Z, Landon BE, Safran DG, Mechanic RE, et al. The Impact of Global Budgets on Pharmaceutical Spending and Utilization. INQUIRY: The Journal of Health Care Organization, Provision, and Financing. 2014 Nov 25; 51(1): 004695801455871. DOI: 10.1177/0046958014558716 PMC4950856 25500751

[113] Donohue JM, Barry CL, Stuart EA, Greenfield SF, Song Z, Chernew ME, et al. Effects of Global Payment and Accountable Care on Medication Treatment for Alcohol and Opioid Use Disorders. Journal of Addiction Medicine. 2018; 12(1): 11–8. DOI: 10.1097/ADM.0000000000000368 29189295 PMC5786473

[114] Blewett LA, Spencer D, Huckfeldt P. Minnesota integrated health partnership demonstration: Implementation of a Medicaid ACO model. Journal of Health Politics, Policy and Law. 2017; 42(6): 1127–42. DOI: 10.1215/03616878-4193666 28801468

[115] Afendulis CC, Hatfield LA, Landon BE, Gruber J, Landrum MB, Mechanic RE, et al. Early Impact Of CareFirst’s Patient-Centered Medical Home With Strong Financial Incentives. Health Affairs. 2017 Mar; 36(3): 468–75. DOI: 10.1377/hlthaff.2016.1321 28264948

[116] Bazzoli GJ. Hospital risk-based payments and physician employment: Impact on financial performance. Health Care Management Review. 2021; 46(1): 86–95. DOI: 10.1097/hmr.0000000000000245 31008806

[117] Lesser CS, Ginsburg PB, Devers KJ. The End of an Era: What Became of the “Managed Care Revolution” in 2001? Health Services Research. 2003; 38(1p2): 337–55. DOI: 10.1111/1475-6773.00119 12650370 PMC1360889

[118] Berwick DM. Payment by Capitation and the Quality of Care. New England Journal of Medicine. 1996 Oct 17; 335(16): 1227–31. DOI: 10.1056/NEJM199610173351611 8815948

[119] Eijkenaar F, Emmert M, Scheppach M, Schöffski O. Effects of pay for performance in health care: A systematic review of systematic reviews. Health Policy. 2013; 110(2–3): 115–30. DOI: 10.1016/j.healthpol.2013.01.008 23380190

[120] Mendelson A, Kondo K, Damberg C, Low A, Motuapuaka M, Freeman M, et al. The effects of pay-for-performance programs on health, health care use, and processes of care: A systematic review. Annals of Internal Medicine. 2017; 166(5): 341–53. DOI: 10.7326/m16-1881 28114600

[121] Wu FM, Shortell SM, Lewis VA, Colla CH, Fisher ES. Assessing Differences between Early and Later Adopters of Accountable Care Organizations Using Taxonomic Analysis. Health Services Research. 2016 Dec; 51(6): 2318–29. DOI: 10.1111/1475-6773.12473 26927979 PMC5134136

[122] Shortell SM, Wu FM, Lewis VA, Colla CH, Fisher ES. A taxonomy of accountable care organizations for policy and practice. Health Services Research. 2014; 49(6): 1883–99. DOI: 10.1111/1475-6773.12234 25251146 PMC4254130

[123] Blackstone EA, Fuhr JP. The Economics of Medicare Accountable Care Organizations. American Health & Drug Benefits. 2016 Feb; 9(1): 11–9. PMC4822974 27066191

[124] Cattel D, Eijkenaar F, Schut FT. Value-based provider payment: towards a theoretically preferred design. Health Economics, Policy and Law. 2018 Sep 27; (2018): 1–19. DOI: 10.1017/S1744133118000397 30259825

[125] Steenhuis S, Struijs J, Koolman X, Ket J, Van Der Hijden E. Unraveling the Complexity in the Design and Implementation of Bundled Payments: A Scoping Review of Key Elements From a Payer’s Perspective. The Milbank Quarterly. 2020 Jan 7; 1468–0009.12438. DOI: 10.1111/1468-0009.12438 PMC7077767 31909852

[126] Liao JM, Pauly MV, Navathe AS. When Should Medicare Mandate Participation In Alternative Payment Models? Health Affairs. 2020 Feb 1; 39(2): 305–9. DOI: 10.1377/hlthaff.2019.00570 32011936

[127] Wong G, Westhorp G, Manzano A, Greenhalgh J, Jagosh J, Greenhalgh T. RAMESES II reporting standards for realist evaluations. BMC Medicine. 2016; 14(1): 1–18. DOI: 10.1186/s12916-016-0643-1 27342217 PMC4920991

[128] Looman W, Struckmann V, Köppen J, Baltaxe E, Czypionka T, Huic M, et al. Drivers of successful implementation of integrated care for multi-morbidity: Mechanisms identified in 17 case studies from 8 European countries. Social Science & Medicine. 2021 May; 277(February): 113728. DOI: 10.1016/j.socscimed.2021.113728 33878666

