# Supplementary material for: The Effect of Network-Level Payment Models on Care Network Performance: A Scoping Review of the Empirical Literature
Source: Int J Integr Care. 2022 Apr 1;22(2):3. doi: 10.5334/ijic.6002 (PMC8973838; doi:10.5334/ijic.6002)
Supplement: Supplementary File. — Search strings. [file ijic-22-2-6002-s1.pdf]

Supplementary file: Search strings

First search:

Database	Before removal duplicates	After
Embase.com	2371	2340
Medline Ovid	455	123
Web of Science Core Collection	1032	513
Cochrane Central Register of Controlled Trials	34	15
**Total**	**3892**	**2991**

Embase.com 2371

('prospective payment'/de OR 'diagnosis related group'/de OR (('economic model'/de OR model/de) AND ('reimbursement'/de OR 'medical fee'/de)) OR 'pay for performance'/de OR 'pay for performance program'/de OR (((prospecti* OR model* OR value-base* OR outcome-base* OR bundle* OR provider* OR reform* OR method* OR scheme* OR system OR systems OR blend* OR virtual*) NEAR/3 (payment* OR reimburse* OR re-imburse* OR purchas* OR remuner* OR fee OR fees OR reward* OR reimburs* OR financing OR funding OR budget OR capitat* OR bonus OR contract OR contracts OR contracting OR contracted OR spending OR pricing)) OR pay-for-performan* OR p4p OR (diagnos* NEAR/3 related NEAR/3 group*) OR drg OR drgs OR (shar* NEAR/3 saving*)):ab,ti) AND ('accountable care organization'/de OR 'health care organization'/de OR (interorganisation* OR inter-organisation* OR interorganization* OR inter-organization* OR (Accountable-Care NEAR/3 (Organization* OR Organisation*)) OR ((care OR clinical OR cancer OR integrated or PROVIDER) NEXT/1 (group* OR network*)) OR (service NEAR/3 deliver* NEAR/3 network*) OR ((health-care OR healthcare* OR integrat*) NEAR/3 network*) OR (integrat*) NEAR/3 (vertical* OR horizon*)):ab,ti) NOT ([Conference Abstract]/lim) AND [English]/lim

Medline Ovid 2371

(Prospective Payment System/ OR Diagnosis-Related Groups/ OR ((Models, Economic/) AND (Reimbursement Mechanisms/ OR Fees, Medical/)) OR Reimbursement, Incentive/ OR pay for performance program/ OR (((prospecti* OR model* OR value-base* OR outcome-base* OR bundle* OR provider* OR reform* OR method* OR scheme* OR system OR systems OR blend* OR virtual*) ADJ3 (payment* OR reimburse* OR re-imburse* OR purchas* OR remuner* OR fee OR fees OR reward* OR reimburs* OR financing OR funding OR budget OR capitat* OR bonus OR contract OR contracts OR contracting OR contracted OR spending OR pricing)) OR pay-for-performan* OR p4p OR (diagnos* ADJ3 related ADJ3 group*) OR drg OR drgs OR (shar* ADJ3 saving*)).ab,ti.) AND (Accountable Care Organizations/ OR "Health Care Economics and Organizations"/ OR Organizations/ OR (interorganisation* OR inter-organisation* OR interorganization* OR inter-organization* OR (Accountable-Care ADJ3 (Organization* OR Organisation*)) OR ((care OR clinical OR cancer OR integrated or PROVIDER) ADJ (group* OR network*)) OR (service ADJ3 deliver* ADJ3

network*) OR ((health-care OR healthcare* OR integrat*) ADJ3 network*) OR (integrat*) ADJ3 (vertical* OR horizon*)).ab,ti.) NOT (news OR congres* OR abstract* OR book* OR chapter* OR dissertation abstract*).pt. AND english.la.

Cochrane CENTRAL register of trials 34

((((prospecti* OR model* OR value-base* OR outcome-base* OR bundle* OR provider* OR reform* OR method* OR scheme* OR system OR systems OR blend* OR virtual*) NEAR/3 (payment* OR reimburse* OR re-imburse* OR purchas* OR remuner* OR fee OR fees OR reward* OR reimburs* OR financing OR funding OR budget OR capitat* OR bonus OR contract OR contracts OR contracting OR contracted OR spending OR pricing)) OR pay-for-performan* OR p4p OR (diagnos* NEAR/3 related NEAR/3 group*) OR drg OR drgs OR (shar* NEAR/3 saving*)):ab,ti) AND ((interorganisation* OR inter-organisation* OR interorganization* OR inter-organization* OR (Accountable-Care NEAR/3 (Organization* OR Organisation*)) OR ((care OR clinical OR cancer OR integrated or PROVIDER) NEXT/1 (group* OR network*)) OR (service NEAR/3 deliver* NEAR/3 network*) OR ((health-care OR healthcare* OR integrat*) NEAR/3 network*) OR (integrat*) NEAR/3 (vertical* OR horizon*)):ab,ti)

Web of science Core Collection 1032

TS=(((((prospecti* OR model* OR value-base* OR outcome-base* OR bundle* OR provider* OR reform* OR method* OR scheme* OR system OR systems OR blend* OR virtual*) NEAR/2 (payment* OR reimburse* OR re-imburse* OR purchas* OR remuner* OR fee OR fees OR reward* OR reimburs* OR financing OR funding OR budget OR capitat* OR bonus OR contract OR contracts OR contracting OR contracted OR spending OR pricing)) OR pay-for-performan* OR p4p OR (diagnos* NEAR/2 related NEAR/2 group*) OR drg OR drgs OR (shar* NEAR/2 saving*))) AND ((interorganisation* OR inter-organisation* OR interorganization* OR inter-organization* OR (Accountable-Care NEAR/2 (Organization* OR Organisation*)) OR ((care OR clinical OR cancer OR integrated or PROVIDER) NEAR/1 (group* OR network*)) OR (service NEAR/2 deliver* NEAR/2 network*) OR ((health-care OR healthcare* OR integrat*) NEAR/2 network*) OR (integrat*) NEAR/2 (vertical* OR horizon*)))) AND DT=(article) AND LA=(english)

Final search:

Database searched	via	Years of coverage	Records	Records after duplicates removed
Embase	Embase.com	1971 - Present	3112	3072
Medline ALL	Ovid	1946 - Present	966	207
Web of Science Core Collection*	Web of Knowledge	1975 - Present	1662	731

Cochrane Central Register of Controlled Trials	Wiley	1992 - Present	44	12
CINAHL	EBSCO	1982 - Present	1073	251
EconLit	ProQuest		96	40
**Total**			**6953**	**4313**

*Science Citation Index Expanded (1975-present) ; Social Sciences Citation Index (1975-present) ; Arts & Humanities Citation Index (1975-present) ; Conference Proceedings Citation Index- Science (1990-present) ; Conference Proceedings Citation Index- Social Science & Humanities (1990-present) ; Emerging Sources Citation Index (2015-present)

Embase.com

('prospective payment'/de OR 'diagnosis related group'/de OR (('economic model'/de OR model/de) AND ('reimbursement'/de OR 'medical fee'/de)) OR 'pay for performance'/de OR 'pay for performance program'/de OR (((prospecti* OR model* OR value-base* OR outcome-base* OR bundle* OR provider* OR reform* OR method* OR scheme* OR system OR systems OR blend* OR virtual* OR population-base*) NEAR/3 (payment* OR reimburse* OR re-imburse* OR purchas* OR remuner* OR fee OR fees OR reward* OR reimburs* OR financing OR funding OR budget OR capitat* OR bonus OR contract OR contracts OR contracting OR contracted OR spending OR pricing)) OR pay-for-performan* OR p4p OR (diagnos* NEAR/3 related NEAR/3 group*) OR drg OR drgs OR (shar* NEAR/3 saving*)):ab,ti,kw) AND ('accountable care organization'/de OR 'health care organization'/de OR (interorganisation* OR inter-organisation* OR interorganization* OR inter-organization* OR (Accountable-Care NEAR/3 (Organization* OR Organisation*)) OR ((care OR clinical OR cancer OR integrated or PROVIDER) NEXT/1 (group* OR network*)) OR ((service OR system*) NEAR/3 deliver* NEAR/3 (network* OR integrat*)) OR ((health-care OR healthcare* OR integrat*) NEAR/3 network*) OR (integrat*) NEAR/3 (vertical* OR horizon*) OR (network NEAR/3 (organisation* OR organization* OR integrat* OR deliver*)) OR (provider* NEAR/3 chain*) OR (health NEAR/3 maintenan* NEAR/3 (organization* OR organization*))):ab,ti,kw) NOT ([Conference Abstract]/lim) AND [English]/lim

Medline Ovid

(Prospective Payment System/ OR Diagnosis-Related Groups/ OR ((Models, Economic/) AND (Reimbursement Mechanisms/ OR Fees, Medical/)) OR Reimbursement, Incentive/ OR (((prospecti* OR model* OR value-base* OR outcome-base* OR bundle* OR provider* OR reform* OR method* OR scheme* OR system OR systems OR blend* OR virtual* OR population-base*) ADJ3 (payment* OR reimburse* OR re-imburse* OR purchas* OR remuner* OR fee OR fees OR reward* OR reimburs* OR financing OR funding OR budget OR capitat* OR bonus OR contract OR contracts OR contracting OR contracted OR spending OR pricing)) OR pay-for-performan* OR p4p OR (diagnos* ADJ3 related ADJ3 group*) OR drg OR drgs OR (shar* ADJ3 saving*)).ab,ti,kf.) AND (Accountable Care Organizations/ OR "Health Care Economics and Organizations"/ OR Organizations/ OR (interorganisation* OR inter-organisation* OR interorganization* OR inter-organization* OR (Accountable-Care ADJ3 (Organization* OR Organisation*)) OR ((care OR clinical OR cancer OR integrated or PROVIDER) ADJ (group* OR network*)) OR ((service OR system*) ADJ3 deliver* ADJ3

(network* OR integrat*)) OR ((health-care OR healthcare* OR integrat*) ADJ3 network*) OR (integrat*) ADJ3 (vertical* OR horizon*) OR (network ADJ3 (organisation* OR organization* OR integrat* OR deliver*)) OR (provider* ADJ3 chain*) OR (health ADJ3 maintenan* ADJ3 (organization* OR organization*))).ab,ti,kf.) NOT (news OR congres* OR abstract* OR book* OR chapter* OR dissertation abstract*).pt. AND english.la.

CINAHL EBSCOhost

(MH Prospective Payment System OR MH Diagnosis-Related Groups OR MH Reimbursement, Incentive OR TI (((prospecti* OR model* OR value-base* OR outcome-base* OR bundle* OR provider* OR reform* OR method* OR scheme* OR system OR systems OR blend* OR virtual* OR population-base*) N2 (payment* OR reimburse* OR re-imburse* OR purchas* OR remuner* OR fee OR fees OR reward* OR reimburs* OR financing OR funding OR budget OR capitat* OR bonus OR contract OR contracts OR contracting OR contracted OR spending OR pricing)) OR pay-for-performan* OR p4p OR (diagnos* N2 related N2 group*) OR drg OR drgs OR (shar* N2 saving*)) OR AB (((prospecti* OR model* OR value-base* OR outcome-base* OR bundle* OR provider* OR reform* OR method* OR scheme* OR system OR systems OR blend* OR virtual* OR population-base*) N2 (payment* OR reimburse* OR re-imburse* OR purchas* OR remuner* OR fee OR fees OR reward* OR reimburs* OR financing OR funding OR budget OR capitat* OR bonus OR contract OR contracts OR contracting OR contracted OR spending OR pricing)) OR pay-for-performan* OR p4p OR (diagnos* N2 related N2 group*) OR drg OR drgs OR (shar* N2 saving*))) AND (MH Accountable Care Organizations OR TI (interorganisation* OR inter-organisation* OR interorganization* OR inter-organization* OR (Accountable-Care N2 (Organization* OR Organisation*)) OR ((care OR clinical OR cancer OR integrated or PROVIDER) N1 (group* OR network*)) OR ((service OR system*) N2 deliver* N2 (network* OR integrat*)) OR ((health-care OR healthcare* OR integrat*) N2 network*) OR (integrat*) N2 (vertical* OR horizon*) OR (network N2 (organisation* OR organization* OR integrat* OR deliver*)) OR (provider* N2 chain*) OR (health N2 maintenan* N2 (organization* OR organization*))) OR AB (interorganisation* OR inter-organisation* OR interorganization* OR inter-organization* OR (Accountable-Care N2 (Organization* OR Organisation*)) OR ((care OR clinical OR cancer OR integrated or PROVIDER) N1 (group* OR network*)) OR ((service OR system*) N2 deliver* N2 (network* OR integrat*)) OR ((health-care OR healthcare* OR integrat*) N2 network*) OR (integrat*) N2 (vertical* OR horizon*) OR (network N2 (organisation* OR organization* OR integrat* OR deliver*)) OR (provider* N2 chain*) OR (health N2 maintenan* N2 (organization* OR organization*)))) NOT PT (news OR congres* OR abstract* OR book* OR chapter* OR dissertation abstract*) AND LA(English)

Cochrane CENTRAL register of trials

((((prospecti* OR model* OR value NEXT base* OR outcome NEXT base* OR bundle* OR provider* OR reform* OR method* OR scheme* OR system OR systems OR blend* OR virtual* OR population NEXT base*) NEAR/3 (payment* OR reimburse* OR re NEXT imburse* OR purchas* OR remuner* OR fee OR fees OR reward* OR reimburs* OR financing OR funding OR budget OR capitat* OR bonus OR contract OR contracts OR contracting OR contracted OR spending OR pricing)) OR pay NEXT for NEXT performan* OR p4p OR (diagnos* NEAR/3 related NEAR/3 group*) OR drg OR drgs OR (shar* NEAR/3 saving*)):ab,ti) AND ((interorganisation* OR inter NEXT organisation* OR interorganization* OR inter NEXT organization* OR (Accountable NEXT Care NEAR/3 (Organization* OR Organisation*)) OR ((care

OR clinical OR cancer OR integrated or PROVIDER) NEXT/1 (group* OR network*)) OR ((service OR system*) NEAR/3 deliver* NEAR/3 (network* OR integrat*)) OR ((health NEXT care OR healthcare* OR integrat*) NEAR/3 network*) OR (integrat*) NEAR/3 (vertical* OR horizon*) OR (network NEAR/3 (organisation* OR organization* OR integrat* OR deliver*)) OR (provider* NEAR/3 chain*) OR (health NEAR/3 maintenan* NEAR/3 (organization* OR organization*))):ab,ti) NOT "conference abstract":pt

Web of science Core Collection

TS=(((((prospecti* OR model* OR value-base* OR outcome-base* OR bundle* OR provider* OR reform* OR method* OR scheme* OR system OR systems OR blend* OR virtual* OR population-base*) NEAR/2 (payment* OR reimburse* OR re-imburse* OR purchas* OR remuner* OR fee OR fees OR reward* OR reimburs* OR financing OR funding OR budget OR capitat* OR bonus OR contract OR contracts OR contracting OR contracted OR spending OR pricing)) OR pay-for-performan* OR p4p OR (diagnos* NEAR/2 related NEAR/2 group*) OR drg OR drgs OR (shar* NEAR/2 saving*))) AND ((interorganisation* OR inter-organisation* OR interorganization* OR inter-organization* OR (Accountable-Care NEAR/2 (Organization* OR Organisation*)) OR ((care OR clinical OR cancer OR integrated or PROVIDER) NEAR/1 (group* OR network*)) OR ((service OR system*) NEAR/2 deliver* NEAR/2 (network* OR integrat*)) OR ((health-care OR healthcare* OR integrat*) NEAR/2 network*) OR (integrat*) NEAR/2 (vertical* OR horizon*) OR (network NEAR/2 (organisation* OR organization* OR integrat* OR deliver*)) OR (provider* NEAR/2 chain*) OR (health NEAR/2 maintenan* NEAR/2 (organization* OR organization*))))) AND DT=(Article OR Review OR Letter OR Early Access) AND LA=(english)

ECONlit ProQuest 60

AB,TI((((prospecti* OR outcome* OR bundle* OR provider* OR reform* OR scheme* OR blend* OR virtual* OR population*) N/3 (payment* OR reimburse* OR purchas* OR remuner* OR fee OR fees OR reward* OR financing OR funding OR budget OR capitat* OR bonus OR contract OR contracts OR contracting OR contracted OR spending OR pricing)) OR "pay-for-performan*" OR p4p OR "diagnos* related group*" OR (shar* N/3 saving*))) AND AB,TI((interorganisation* OR interorganization* OR inter-organization* OR "Accountable-Care Organization*" OR "Accountable-Care Organisation*" OR ((care OR clinical OR cancer OR integrated OR provider) N/1 (group* OR network*)) OR ((health-care OR healthcare* OR integrat*) N/3 network*) OR (integrat*) N/3 (vertical* OR horizon*) OR (network N/3 (organisation* OR organization* OR integrat* OR deliver*)) OR (provider* N/3 chain*)))
